# Impact of Antibiotics as Waste, Physical, Chemical, and Enzymatical Degradation: Use of Laccases

**DOI:** 10.3390/molecules27144436

**Published:** 2022-07-11

**Authors:** María P. C. Mora-Gamboa, Sandra M. Rincón-Gamboa, Leidy D. Ardila-Leal, Raúl A. Poutou-Piñales, Aura M. Pedroza-Rodríguez, Balkys E. Quevedo-Hidalgo

**Affiliations:** 1 Laboratorio de Biotecnología Molecular, Grupo de Biotecnología Ambiental e Industrial (GBAI), Departamento de Microbiología, Facultad de Ciencias, Pontificia Universidad Javeriana, Bogotá 110-23, Colombia; mora_mariapaula@javeriana.edu.co (M.P.C.M.-G.); sandra_rincon@javeriana.edu.co (S.M.R.-G.); 2 Laboratorio de Microbiología de Alimentos, Grupo de Biotecnología Ambiental e Industrial (GBAI), Departamento de Microbiología, Facultad de Ciencias, Pontificia Universidad Javeriana, Bogotá 110-23, Colombia; 3 Laboratorio de Biotecnología Vegetal, Grupo de Investigación en Asuntos Ambientales y Desarrollo Sostenible (MINDALA), Departamento de Ciencias Agrarias y del Ambiente, Universidad Francisco de Paula Santander, Ocaña 546552, Colombia; 4 Laboratorio de Microbiología Ambiental y de Suelos, Grupo de Biotecnología Ambiental e Industrial (GBAI), Departamento de Microbiología, Facultad de Ciencias, Pontificia Universidad Javeriana, Bogotá 110-23, Colombia; apedroza@javeriana.edu.co; 5 Laboratorio de Biotecnología Aplicada, Grupo de Biotecnología Ambiental e Industrial (GBAI), Departamento de Microbiología, Facultad de Ciencias, Pontificia Universidad Javeriana, Bogotá 110-23, Colombia; bquevedo@javeriana.edu.co

**Keywords:** antibiotics, laccases, wastewaters, antimicrobial resistance, treatment

## Abstract

The first traces of Tetracycline (TE) were detected in human skeletons from Sudan and Egypt, finding that it may be related to the diet of the time, the use of some dyes, and the use of soils loaded with microorganisms, such as *Streptomyces* spp., among other microorganisms capable of producing antibiotics. However, most people only recognise authors dating between 1904 and 1940, such as Ehrlich, Domagk, and Fleming. Antibiotics are the therapeutic option for countless infections treatment; unfortunately, they are the second most common group of drugs in wastewaters worldwide due to failures in industrial waste treatments (pharmaceutics, hospitals, senior residences) and their irrational use in humans and animals. The main antibiotics problem lies in delivered and non-prescribed human use, use in livestock as growth promoters, and crop cultivation as biocides (regulated activities that have not complied in some places). This practice has led to the toxicity of the environment as antibiotics generate eutrophication, water pollution, nutrient imbalance, and press antibiotic resistance. In addition, the removal of antibiotics is not a required process in global wastewater treatment standards. This review aims to raise awareness of the negative impact of antibiotics as residues and physical, chemical, and biological treatments for their degradation. We discuss the high cost of physical and chemical treatments, the risk of using chemicals that worsen the situation, and the fact that each antibiotic class can be transformed differently with each of these treatments and generate new compounds that could be more toxic than the original ones; also, we discuss the use of enzymes for antibiotic degradation, with emphasis on laccases.

## 1. Introduction

Antimicrobials are drugs that had great success in the history of medicine; their discovery is associated frequently with the modern era after great scientists, such as Paul Ehrlich and Alexander Fleming. Ehrlich, in 1904, discovered Salvarsan; the first antibacterial drug used to treat syphilis in the world, this drug had undesirable side effects, so Salvarsan was replaced later by Penicillin (P) in 1940. Alexander Fleming discovered Penicillin (P) in 1928 when noticed that the fungus *Penicillium notatum* inhibited the growth of Staphylococcus. Gerhard Domagk, in 1935, demonstrated that Prontosil (the first sulphonamide drug) was effective against Streptococcus infections and puerperal fever [1,2].

The first traces of Tetracycline (TE) detected in humans were in skeletons from Nubia (350–550 BC) in Sudan and Egypt (in the Dakhleh Oasis, late Roman period), probably associated with the consumption of foods containing producing microorganisms or traces of the antibiotic [1]. However, Penicillin (P) resistance appeared first in 1940 and later in 1948.

Streptomycin (STR) resistance was reported. Since then, many research and scientific developments have accompanied the treatment of infectious diseases, including the synthesis of cephalosporins [2].

Today antibiotics are the second most common group of drugs found in wastewaters worldwide because they are widely used in livestock, as prophylactics, and as a treatment for diseases caused by pathogens (metaphylactic); allowing healthy growth, lower morbidity, and mortality [3,4]. Antibiotics are used in agriculture to combat diseases, as biocides in crop and fruit production, or as a growth factor in the rearing of animals intended for meat, milk, or egg production, such as pigs, cattle, poultry, and fish [5]. The use of antibiotics in crops is not enough documented; however, the use in this area is more problematic for the environment, as the tonnes of Streptomycin (STR) used for crops can be up to 292 tonnes compared to 6.3 tonnes used annually for American medicine [6].

According to a report by the Food and Drug Administration (FDA), the number of antimicrobial drugs (medically relevant) approved for use in food-producing animals and actively marketed from 2011 to 2020 in the United States of America ranged from 6,002,056 to 9,702,943 total annual (Kg), with a notable reduction in 2018, 2019 and 2020 (6,032,298; 6,189,260 and 6,002,056, respectively). On the other hand, the use of antimicrobial drugs (not medically relevant) ranged between 4,447,420 and 5,882,221 annual total (Kg), also with a significant decrease in the last three years (5,530,784; 5,279,098; 4,447,420, respectively), which shows the great effort made to control the use of these molecules [7].

Due to antibiotics’ nature, some of them are poorly absorbed by the gut of the animals, in general, resulting in 30 to 90% excretion of the parent compound [8]. Most antibiotics are water-soluble, which means that up to 90% of the dose administered to the animal can be excreted in the urine and up to 75% in the faeces. When livestock waste is disposed of, the antibiotics in faeces are usually decomposed due to oxidation–reduction reactions, hydrolysis, biodegradation, and photodegradation, which reduces the concentration of the antibiotic and thus its bactericidal or bacteriostatic function [9]; this generates a problem, as the exposure of microorganisms to sub-lethal concentrations, favours the adaptation and proliferation of bacteria with multi-resistant phenotypes. In addition, antibiotics generate a serious environmental contamination problem, harming the ecosystems due to their toxicity in aquatic organisms, such as algae, bacteria, and fish [10].

On the other hand, bacterial resistance to antibiotics leads to treatment failure, the chronic behaviour of infectious processes, the progression of the disease from acute to chronic, the transmission of the infection to other animals, outbreaks, economic losses and even the appearance of adverse reactions to antibiotics [11]. In many cases, crops for human consumption are fertilised with animal faeces and irrigated with antibiotic-contaminated water sources, resulting in the spread of resistant strains to humans and animals. This spread generates a public health problem, as treatment options against pathogenic bacteria are becoming increasingly limited [12].

There are different methods for the removal of pharmaceutical compounds from wastewaters; however, due to the hydrophilicity, persistent nature, and low concentration in wastewaters, the removal of these chemicals in conventional wastewaters treatment plants is much more laborious than the removal of other organic matter and nutrients. Some processes result in primary and tertiary sludge with high concentrations of micropollutants [13], which need managing by using other types of treatments, such as incineration or plasma melting, which considerably increases treatment costs [14]. In addition, oxidation processes can produce antibiotic degradation intermediates that are toxic and carcinogenic [15,16]. Therefore, environmentally friendly and efficient alternatives for the degradation of these compounds have been sought to reduce the problem of contamination and bacterial resistance.

## 2. Antibiotics

Antibiotics are natural or synthetic molecules capable of inhibiting the growth of bacteria (bacteriostatic) or killing some bacteria (bactericidal). Antibiotics exert a specific action on certain structures or functions of the microorganism. They are classified according to their mechanism of action: inhibitors of cell wall formation, inhibitors of protein synthesis, inhibitors of DNA replication, inhibitors of cytoplasmic membrane synthesis and inhibitors of metabolic pathways [17]. Additionally, they can be classified according to their action spectrum as a broad- or limited-spectrum, and according to their pharmacokinetics, concerning gastrointestinal, subcutaneous or muscular absorption, distribution and elimination (hepatic or renal) in the human or animal body [5]. Table 1 shows the different antibiotics of critical importance and high importance, according to the WHO (World Health Organization) [5,8,18,19,20]. The WHO classification meets two criteria, first, if the antimicrobial class is the only, or one of the few, available to treat human infections, second, the antimicrobial class is for treating human infections caused by (i) bacteria transmitted from non-human sources, (ii) bacteria that can acquire resistance genes from non-human sources. If both criteria are fulfilled, the antibiotic is critically important; on the other hand, if only one criterion is met, the antibiotic is highly important [21,22].

Within the critical importance category, aminoglycosides (Table 1) are commonly used in clinical practice. Various resistance mechanisms have been reported; however, these compounds are active against most aerobic Gram-negative bacilli, as well as the β-lactams. Due to their polycationic nature, aminoglycosides are highly soluble in water and exhibit low gastrointestinal absorption, resulting in their excretion into the environment. Additionally, due to their chemical structure, these antibiotics are stable. The presence of these compounds in water and soil causes ecotoxicity; for example, Streptomycin (STR) residues are toxic to algae [36]. Wollenberger et al. (2000) performed biotoxicity tests of Streptomycin (STR) against *Daphnia magna*, obtaining a value of the median effective concentration of about 48 h EC50 = 487 mg L^−1^ [37]. Furthermore, Streptomycin (STR) resistance genes have been identified in pathogenic bacteria, such as *Salmonella* spp., and *Enterococcus* spp., [38,39]. In addition to aminoglycoside’s ecotoxicity, they can cause adverse effects in humans because they are ototoxic and nephrotoxic, and cause neuromuscular toxicity (less frequently), toxicity in the haematopoietic system, and also allergic reactions (rash, fever, angioedema or exfoliative dermatitis). However, the risk of toxicity depends on different factors, such as pre-existing diseases, the severity of the disease, concomitant drugs, and genetic predisposition [40,41].

Ansamycins are considered critically important, these antibiotics are used as sole or limited therapy against tuberculosis. Rifampicins serve to treat several types of mycobacterial infections, such as *Mycobacterium avium* complex, and leprosy, and combined with other antibacterials, allow for treating latent or active tuberculosis. Up to 50% of this antibiotic class is excreted in faeces, affecting the environment. This antibiotic class has been found in water, and it is known for its cytotoxic effects [21,42,43].

β-Lactam antibiotics also fall into this category. They make up the majority of antibiotics in human use in most countries and account for approximately 50 to 70% of antibiotics used [44]. Compared to other compounds, they are not stable in the environment as the β-lactam ring can be degraded by β-lactamase (E.C 3.5.2.6), a common enzyme in several bacteria, and can also be degraded by chemical hydrolysis [44]. Despite the above, the presence of β-lactams and β-lactamase (E.C 3.5.2.6) in wastewaters, responsible for bacterial resistance to these antibiotics, has been demonstrated [45]. For example, Hoelle et al. (2019) isolated 332 *E. coli* strains in which 65 (19.6%) were resistant to carbapenems [46]. Toxicity bioassays by Havelkova et al. (2016), evaluated the toxicity of Penicillin G (PG) against *Daphnia magna*, obtaining values of 48 h EC50 = 1496.9 mg L^−1^ [44]. On the other hand, the adverse effects of penicillins and cephalosporins are hepatotoxicity, and can cause neutropenia and encephalopathy; these antimicrobials also cause neuronal excitation because the β-lactam ring is structurally similar to the neurotransmitter GABA inhibitor, causing inhibition of the gamma-aminobutyric acid (GABA) system [47]. Among the β-lactams are the cephalosporins, a broad-spectrum antibiotic class used against Gram-positive and Gram-negative causing infections. According to their activity and spectrum, cephalosporins are classified into five generations [48]. Cephalosporins are useful for both humans and animals, and approximately more than 50% of these drugs are eliminated structurally unmodified through the urine [49]. Cephalosporins are the most produced, prescribed, and consumed class of antibiotics in Europe, and their presence in wastewaters represents a problem, as they contribute to increased COD (Chemical Oxygen Demand); increasing remediation costs [48]. High concentrations of cephalosporins have also been reported ranging from μg L^−1^ to mg L^−1^ [50,51]. Penicillins are broad-spectrum β-lactams for infectious diseases treatment in humans and animals; however, the emergence of multiresistant β-lactamase (E.C 3.5.2.6) or penicillinase (E.C 3.5.2.6) producing bacteria has limited the use of penicillins in recent years. Penicillins are still recommended and widely used by physicians [52], but a dose of penicillin is generally excreted through the urine without structural changes; however, they could be transformed [53]. The environmental presence of these compounds has been found in concentrations of about 1 to 1000 μg L^−1^ in aquatic environments [54]. Penicillins are ecotoxic; the presence of penicillins in untreated effluents can cause the death of *Daphnia magna* [55].

On the other hand, glycopeptides are antibiotics considered of critical importance and detected in high concentrations in wastewaters [56,57], generating a harmful environmental impact. Havelkova et al. (2016) developed biotoxicity bioassays for vancomycin, obtaining against *D. magna,* values of 48 h EC50 = 686.9 mg L^−1^ [44]. The presence of glycopeptides in aquatic environments represents a problem due to antimicrobial resistance. For several years, different pathogenic species of the genus Enterococcus have been isolated from livestock farms and classified as resistant to Vancomycin (VA) and Teicoplanin (TEC). Likewise, other bacteria causing infections in humans, such as *Staphylococcus aureus*, have unfortunately acquired resistance to commonly used antibiotics, including Vancomycin (VA) [58].

Tigecycline (TGC), a critically important antibiotic, is used as a limited therapy for infections caused by multidrug-resistant Enterobacteriaceae and methicillin-resistant *Staphylococcus aureus* [21,22,43]. It is used to treat infections, such as community-acquired pneumonia, complicated intra-abdominal infections, and complicated skin infections [59]. 59% of the Tigecycline (TGC) dose is excreted in the biliary/faecal matter, and 33% is excreted in the urine; 22% of the drug is structurally unchanged; which has increased bacterial resistance to Tigecycline (TGC). The presence of the *Tet*X gene is responsible for Tigecycline (TGC) resistance and has been detected in aquatic environments and wastewaters treatment systems [60].

Lipopeptides are of critical importance to antibiotics; especially the use of polymyxins decreased due to their adverse effects (nephrotoxicity); however, because of the emergence of multi-resistant Gram-negative bacteria and the lack of new antibiotics, they are increasingly used as a last-line treatment against these pathogens. According to Zurfuh et al. (2016), there is no literature about polymyxins detection in water, attributed to the lack of extraction methods; however, bacteria with polymyxin resistance genes have been found in wastewaters, surface water and groundwater [61,62,63].

Macrolides are also of critical importance and are a limited therapy against *Legionella* spp., *Campylobacter* spp., multidrug-resistant *Salmonella* spp., and *Shigella* spp., [21,22,43,64]. Macrolides have high activity against Gram-positive and Gram-negative bacteria in humans and animals. Up to 50% of macrolides are excreted in faeces; they have been detected in water (varying concentrations) [65,66], and their cytotoxic effects have been proven [67].

Oxazolidinones are of critical importance and a limited therapy against multidrug-resistant *Staphylococcus aureus* and *Enterococcus* spp., infections [21,22,43]. They are also considered “last-resort” antibiotics, which are important against Gram-positive bacteria resistant to β-lactams and glycopeptides. However, resistance genes have been detected in pathogens, such as *Enterococcus* spp., and *Clostridium perfringens* [68,69]. In addition, the toxicity of this class of antibiotics and their degradation products has been demonstrated [70,71].

Fosfomycin (FOS) also belongs to antibiotics of critical importance and essential medicines [72]. It is used as a limited therapy against extended-spectrum β-lactamase (E.C 3.5.2.6) (ESBL)-producing *E. coli*, which causes urinary tract infections [21,22,43]. In poultry, it is used in infectious diseases, because it is effective against bacteria, such as *E. coli*, *Klebsiella* spp., *Proteus mirabilis*, *Staphylococcus saprophyticus*, *Enterococcus* spp., and *Streptococcus agalactiae* [73]. Fosfomycin (FOS) is excreted without structural change approximately 38% in urine, while 18% in faeces [74]. The presence of Fosfomycin (FOS) in wastewaters from the pharmaceutical industry and the presence of fosfomycin resistance genes have been reported. The high concentrations of FOS make them recalcitrant and toxic to the environment, as they can trigger eutrophication leading to water pollution and nutrient imbalance [75,76,77].

Quinolones are considered critically important and used as a limited therapy against *Campylobacter* spp., the invasive disease caused by *Salmonella* spp., the multidrug-resistant *Salmonella* spp., and *Shigella* spp., [21,22,43,64]. They are used for the treatment of diseases in both humans and animals. Due to their high adsorption potentials, non-degraded compounds in the treatment plants are adsorbed into the sludge in small fractions; subsequently being released into the environment. Molecules, such as Ofloxacin (OFL), Ciprofloxacin (CIP), Nalidixic acid (NAL), Norfloxacin (NX), and resistance genes have been detected in wastewaters. The toxicity of these compounds and adverse effects in humans have also been demonstrated, with gastroenteritis and joint pain being the most common effects [78,79,80,81,82].

Phenicols are broad-spectrum antibiotics for infectious disease treatment in both humans and animals. It is a high importance antibiotic class [21,22,43]. In aquaculture, Florfenicol (FLO) and Chloramphenicol (CHL) are frequently used although their veterinary use is restricted [83]. Furthermore, this antimicrobial can be excreted without structural changes, increasing its presence in the environment.

On the other hand, lincosamides are a highly important antibiotics class, used against infections caused by *Enterococcus* sp., and *Staphylococcus aureus*. Due to the reduced susceptibility of *S. aureus* to β-lactam antibiotics, the clinical use of macrolides and lincosamides against penicillin-resistant *S. aureus* has been promoted [84]. Bruyndonckx et al. (2021) reported that lincosamides were among the most consumed classes in Sweden and accounted for more than 20% of macrolides, lincosamides and streptogramins consumption in Austria, Finland, Germany, Hungary, and Luxembourg between 1997 and 2017 [52]. Lincosamide resistance genes have been reported in pharmaceutical industry wastewaters treatment plants [85].

Pseudomonic acids have a unique chemical structure and mode of action, are highly relevant, have a broad spectrum of activity against Gram-positive bacteria, and exhibit “in vitro” activity against some Gram-negative bacteria [86]. They are mainly used to treat impetigo caused by *Staphylococcus aureus* and *Streptococcus pyogenes*; however, mupirocin-resistant *S. aureus* has already been reported [86].

Riminophenazine (RPZ) is a high importance antibiotic for treating dapsone-resistant lepromatous leprosy and lepromatous leprosy complicated by erythema nodosum leprosum; Riminophenazine (RPZ) is used in combination with other compounds, such as dapsone. Clofazimine (CFZ) is an antimicrobial dye that produces the discolouration of skin and body fluids and has “in vitro” activity against mycobacteria, such as *Mycobacterium tuberculosis*. However, it is ineffective compared to classical anti-TB treatments, such as Rifampicin (RIF) and Isoniazid (INH). After a dose of 300 mg, the concentration in urine and faeces is negligible Holdiness [87].

Fusidic acid (FUS) is also of high importance antibiotic [21,22,43], used topically to prevent and treat skin infections. It is widely used in Europe and has activity against Gram-positive bacteria. The resistance of *S. aureus* strains to Fusidic acid (FUS) has been also demonstrated [88].

Sulphonamides are also of high importance [21,22,43], with a broad spectrum used for the treatment of infectious diseases in both humans and animals (in combination with other compounds). Some time ago, these antibiotics were widely used in humans; however, their adverse effects and the emergence of resistant bacteria, have led to restrictions on their human use; in contrast, are widely used in pigs and cattle. The main releasing route for these antibiotics into the environment is farm wastewaters. This class of compounds has been detected in effluents from treatment plants, surface water, and groundwater. Genes conferring resistance to these antibiotics have also been identified [89,90].

Tetracyclines are of high importance main class of antibiotics against infectious diseases in humans and livestock. They are broad-spectrum and low-cost antibiotics, but their hydrophilic nature, favour more than 75% of the dose being excreted, without structural changes [91]. These antibiotics have been detected in water, sludge, and various aquatic environments combined with cations (Ca^2+,^ Mg^2+^ y Cu^2+^) [92]. Sixty-five resistance genes of first- and second-generation tetracyclines are distributed among 130 species of Gram-negative and Gram-positive microorganisms, including *Mycobacterium* spp., and *Streptomyces* spp., [93].

## 3. Antibiotic Use in Livestock Farming

Antibiotics are frequent in the livestock industry to prevent and treat diseases caused by bacteria, allowing for healthy growth and reduced animal mortality and morbidity. The use of these antibiotics occurs principally in pigs, cattle, poultry, and the aquaculture industry [5,94] Livestock farmers use antibiotics for metaphylactic, prophylactic, and growth promotion purposes. Metaphylaxis is the administration of drugs to presumably infected or disease susceptible groups of animals to treat and control the disease transmission among animals in close contact. Prophylaxis is the administration of drugs to a group of animals before clinical signs of a disease to prevent its occurrence [95]. Antibiotics also can be used as growth promoters; the destruction of the intestinal microbiota generates increased assimilation of the feed consumed; therefore, an increase in the muscle mass of the cattle occurs fewer times; however, this practice has been banned in some countries [11].

Globally, cattle account for 34% of the world’s dietary protein supply. In 2019, China was the largest consumer of beef, poultry, and pork (71,338 thousand tonnes), followed by the European region (39,862 thousand tonnes), the United States (39,225 thousand tonnes), Brazil (20,956 thousand tonnes), and Russia (9894 thousand tonnes). It has been estimated that 261.906 million tonnes of meat (beef, pork, and chicken) were consumed worldwide in 2019 (https://comecarne.org accessed on 12 April 2022). In Colombia, by 2020, there were 28,245,262 cattle head, 221,011 pigs head, and 463,113 poultry head (https://www.ica.gov.co/areas/pecuaria/servicios/epidemiologia-veterinaria/censos-2016/censo-2018.aspx accessed on 12 April 2022).

According to a report by the FDA between 2009 and 2018, the annual amount of antibiotics used in animals reached 8,000,000 Kg, with tetracyclines being the most widely used [7]. Global consumption of antimicrobials used in food-producing animals was estimated at 63,151 tonnes (http://www.fao.org/antimicrobial-resistance/key-sectors/animal-production/en/ accessed on 12 April 2022).

### 3.1. Antibiotics Use in Livestock Farming and Foodborne Diseases (FBD)

According to the WHO, Foodborne Diseases (FBD) are diseases commonly transmitted through the ingestion of contaminated food. FBD involves a broad group of illnesses caused by microbial pathogens, parasites, chemical contaminants, or biotoxins [96]. The severity of these diseases in humans varies from mild to severe symptoms, which require lifelong treatment. It has been estimated that, in industrialised countries, more than 10% of the population could suffer from a disease associated with the ingestion of contaminated food [97]. One of the aspects that increase the severity of FBD is the presence of antibiotic-resistant microorganisms, leading to therapeutic failure in humans. This resistance is directly associated with the use of antimicrobials for prophylaxis, metaphylaxis, or as growth factors during livestock destinated to a source of meat, eggs or milk [98].

In animal food production, the use of antibiotics has increased; for example, 63,151 tons of antibiotics were used in livestock production in 2010, and have been estimated that there will be a 67% increase by 2030 [99].

The scenario tends to be worsened by manure used as a crop fertiliser, leading to increased dissemination of bacteria and antibiotic resistance genes. Disseminated bacteria and resistance genes will have contact with the soil microbiome, which could lead to loss of biomass and a reduction in nitrification, denitrification, and respiration activity, as well as impairment of enzyme activity, such as dehydrogenases (E.C. 1.1.-), phosphatases (E.C. 3.1.3.-), phenoloxidase (E.C. 1.10.3.), ammonium monooxygenase (E.C. 1.14.99.39), and ureases (E.C. 3.5.1.5), considered important indicators of soil activity. The exact amount of antibiotic use in animal production is difficult to define; however, it is higher than hospital use [99,100].

Pathogen entry into the meat supply chain occurs at any stage of production (rearing, processing, distribution, sale, handling, and preparation) [101]; some authors have described the slaughter stage, during the removal of the gastrointestinal tract or intestinal contents, as the principal source of pathogen dissemination by cross-contamination through equipment, utensils and personnel [101,102,103,104,105], increasing the risk of contamination at the production line, considering the ability of some of the microorganisms (Enterobacteria) to generate biofilms [106].

The entry of antibiotics into the meat supply chain occurs in feeding operations, where concentrates contain antibiotics used as growth promoters. However, some EU countries have prohibited antibiotics use as growth promoters (since 2006) because they applied for prophylaxis or metaphylaxis [99,100].

On the other hand, in the meat processing stage, antimicrobial resistances are transmitted through the entry of food contaminated with resistant microorganisms or through the food processing environment. Mentioning a couple of microorganisms, *Salmonella* spp., and *Listeria monocytogenes* are two of the most important bacterial agents in FBD.

*Salmonella* spp., (one of the pathogens transmitted during the slaughter of livestock) is the causing agent of salmonellosis, a disease that has a high morbidity rate in both industrialised and developing countries; causing acute enterocolitis with abdominal pain, bloody or non-bloody diarrhoea, nausea, and vomit [64,106,107]. Transmission of non-typhoidal Salmonella to humans can occur zoonotically through contact with faecal material from carrier animals or by the consumption of contaminated food [64,106,107,108,109].

On the other side, *L. monocytogenes* (one of the pathogens transmitted during the production of processed foods) is the causative agent of listeriosis, an invasive disease; therefore, needs to cross through the intestinal barrier to gain access to internal organs, the entry takes place through Peyer’s patches by M-cells. Subsequently, the bacterium goes to the liver and may develop granulomatous hepatitis, due to the invasion of hepatocytes. Afterward, the bacterium is internalized, and, in some cases, there is intracellular proliferation and dissemination to other tissues, with tropism for the Central Nervous System (CNS) and the pregnant uterus [110,111,112,113,114].

In developing countries, the main route of contamination is through consumption of contaminated vegetables, contaminated water and human-human contact, whereas in industrialised countries, the major route of contamination is related to ingestion of contaminated food products of animal origin, especially fresh meat and eggs [64,106].

The final food consumer comes into contact with antibiotic-resistant microorganisms through their presence in ready-to-eat meat, dairy and vegetable products [115]. Contact with microorganisms carrying antibiotic resistance genes can lead to two possible scenarios: (i) the modification of the gut microbiome [100], (ii) a potential hazard to human medicine due to the inability to manage infections in the general population, resulting in prolonged broad-spectrum antibiotic treatments, which would end up in a constant loop between resistances going into the environment and the population [99,100].

### 3.2. Waste from the Livestock Industry

According to the United States Department of Agriculture (USA), animals confined for food production generate approximately 335 million tonnes of waste per year, exceeding 40 times the mass of biosolids generated by humans [5,8].

The principal cause of bacterial resistance to antibiotics is the disposal of antibiotics in livestock waste in soil, ground and surface water, atmosphere and crops [5,9]. When livestock wastes are disposed of, the antibiotics in faeces are often broken down due to oxidation–reduction reactions, hydrolysis, biodegradation and photodegradation, reducing the concentration of the antibiotic and thus its ability to kill bacteria [116]; which generates a problem, as bacterial exposure to sub-lethal concentrations; favouring the adaptation and proliferation of bacteria with multidrug-resistant phenotypes. Whereas a high proportion of excreted antibiotics are bioactive, consequently, and due to fast bacterial reproduction, the resistance phenotype can easily express Sarmah [8].

Bacteria can resist antibiotics through several mechanisms; through physiological adaptation, bacteria can change membrane transport pumps (porins), excluding harmful agents; however, this mechanism is limited Silbergeld [5]. Another mechanism is mutations, which can alter or eliminate the target of the antibiotic [116], mutations could occur in DNA sequences that modify the production or structure of the enzymes responsible for inactivating the antimicrobial agent; for example, β-lactamases (E.C. 3.5.2.6) can inactivate the β-lactam ring, which is part of the structure of β-lactam antibiotics (Table 1). These enzymes break the amide bonding of the ring, and the generation of acidic derivatives that do not have antibacterial properties occurs by preventing the antibiotics from binding to bacterial carrier proteins [117].

Resistance genes can be shared between bacteria by three different mechanisms (transformation, conjugation, and transduction), thus transferring resistance between pathogenic and non-pathogenic bacteria, this transfer is called “horizontal transfer” and can occur between bacteria of different genera. Vertical transfer occurs during cell division through the genetic material transmission from mother to daughter cell [118].

Conjugation is one of the most effective mechanisms for genetic material transfer and is considered one of the reasons for antibiotic resistance. Conjugation is frequent in Gram-negative bacteria and to a lesser extent in Gram-positive bacteria [119,120]. During the Gram-negative conjugation process, a donor bacterium transfers a plasmid containing resistance genes to a recipient bacterium throughout sexual pilis [120,121]. During the Gram-positive conjugation process, the receptor cells excrete an inducing peptide called pheromones or autoinducers. When a certain level of peptide concentration is reached the donor cell activates the transcription of genes related to the production of aggregation substances, including adhesins and coupling proteins. The donor cell attaches to the receptor cell, which generates a channel to transfer the genetic material [122].

The transduction process, allows resistance genes to be transferred from one bacterium to another mediated by a bacteriophage that has previously undergone lytic cycling in the “donor strain”.

The transformation process occurs when a bacterium incorporates DNA from the environment favoured by the state of competence generated during cell growth [116]. Furthermore, mobile genetic elements, such as plasmids, insertions sequences, transposons, and integrons are responsible for 95% of antibiotic resistance [5].

Bacterial resistance to antibiotics implies the treatment’s failure against infections, the latency of infectious processes, progression of the disease to chronic processes, the transmission of the infection to other animals, outbreaks, economic losses, and even adverse reactions to antibiotics [11]. In many cases, crops for human consumption are fertilised with animal faeces and irrigated with water sources contaminated with antibiotics, resulting in the spread of resistant strains to humans and animals. Spread generates a public health problem, as treatment options against pathogenic bacteria are limited [12].

In the last 20 years, the presence of antibiotics has been documented in different water sources; surface, ground, and oceanic waters [15,123]. In addition to water sources, food contaminated with antibiotics can cause adverse allergic reactions in hypersensitive individuals [11]. Ramírez et al. (2012) administered six antibiotics intramuscularly and intramammary to 115 cows and found that 65.2% of cows had Oxytetracycline (OXT), 58.8% of cows had Tylosin (TLY), 69.2% of cows had Spiramycin (SP) and 65% of cows had Amoxicillin (AMC) traces in milk [124].

The presence of antibiotics in water is a problem for ecosystems; Xu et al. (2019) demonstrated the toxicity of Tetracycline (TE) and its degradation intermediates (anhydrotetracycline and epitetracycline hydrochloride) in microalgae. At high concentrations of Tetracycline (TE) (>5 mg L^−1^), the permeability of microalgae is affected, showing structural changes and increasing reactive oxygen species (ROS) activity in the water [125].

## 4. Antibiotic Use by Humans

Humans consume a large variety of antibiotics [95], and the consumption rate per class differs between countries; according to the WHO antibiotic consumption monitoring report, in 2015, Mongolia was the highest consumer of antibiotics among East and South-East Asian countries, reaching approximately 65 defined daily doses (ddd) per 1000 population. In this country, the highest consumption involved penicillins and other β-lactams. Other countries in the East and South-East Asia region also participated in the surveillance of antibiotic consumption; the Republic of Korea (27 ddd), New Zealand (23 ddd), Japan (15 ddd), Philippines (8 ddd) and Brunei Darussalam (5 ddd). Among these six countries, Amoxicillin was the most widely consumed [43]. On the other hand, in China, the percentage of human prescriptions (between 41 and 61%) exceeds the WHO recommended threshold (30%). China is considered the world’s largest consumer of antibiotics for animal and human use; in 2011, the annual per capita antibiotic use was 138 g, exceeding ten times that of the United States [126]. Some authors attribute this high consumption to different factors. (i) self-medication, (ii) the large proportion of patients receiving combination therapy with multiple antibiotics, (iii) inappropriate antibiotic management in health care facilities and pharmacies (especially in rural areas), (iv) and a lack of public understanding and awareness of antibiotic use [126,127]. East and South-East Asian countries have made efforts to prevent inappropriate use of antibiotics and antibiotic resistance; however, there are still some gaps, such as the lack of guidelines for antibiotic residue disposal, lack of environmental standards and lack of monitoring databases [127].

Countries belonging to the Eastern Mediterranean region also reported antibiotic consumption; countries, such as Iran, Jordan and Sudan reported 38, 8 and 35 ddd per 1000 inhabitants, respectively. The highest consumption in 2015 was for penicillins, other beta-lactams, macrolides and tetracyclines [43].

Similarly, African countries, such as the United Republic of Tanzania, Côte d’Ivoire, Burundi and Burkina Faso reported consumption of 27, 11, 4 and 14 ddd per 1000 population in 2015, respectively. In these countries, penicillins, tetracyclines, sulphonamides and quinolones were the most consumed [43].

Countries of the European Centre for disease prevention and control implemented a surveillance system for antibiotic consumption in the primary and hospital sector. As a result, since 1997, 31 countries have usually reported consumption data expressed as ddd per 1000 population. The highest consumption has been of beta-lactams, with penicillins being the main antibiotics consumed (https://www.ecdc.europa.eu/en/antimicrobial-consumption/database/country-overview accessed on 27 April 2022). Some European countries also participated in the WHO surveillance system in 2015. Reports from countries, such as Turkey (37 ddd), Serbia (32 ddd), Greece (28 ddd), Romania (29 ddd), Montenegro (27 ddd), Italy (27 ddd), France (26 ddd), and Belgium (25 ddd) were the highest. Penicillins, other β-lactams, macrolides and tetracyclines were the most consumed [43].

Some countries in the Americas participated in the WHO report; Bolivia, Brazil, Canada, Costa Rica, Paraguay, and Peru reported consumption of 19, 23, 17, 14, 18, and 11 ddd per 1000 inhabitants, respectively, with penicillins being the most consumed, followed by other β-lactams, macrolides and quinolones [43]. The United States has a surveillance system for antibiotic consumption, with approximately 200,000 antibiotic prescriptions dispensed in retail pharmacies reported in 2020 [128].

In Latin America, surveillance and records of antibiotic consumption in humans are scarce. In Colombia, there are some institutions responsible for surveillance and control of human consumption of antibiotics; the National Institute of Health published a report on antibiotic consumption in the hospital setting between 2013 and 2017; monitoring antibiotics, such as Ceftriaxone (CRO), Imipenem (IPM), Meropenem (MEM), Piperacillin-Tazobactam (PIP-TZB) Vancomycin (VA) and Ciprofloxacin (CIP); with Meropenem (MEM) and Ciprofloxacin (CIP) being the most consumed [129]. Some studies have implemented surveillance systems; Pallares et al. (2011) implemented a program for the regulated use of antibiotics at the Hospital Universitario del Valle, achieving an important reduction in the consumption of antibiotics in two intensive care units (ICU) and infection by resistant microorganisms [130]. In the study, there was a significant reduction in the consumption of antibiotics, such as ceftriaxone (ICU-1 *p* = 0.015 and ICU-2 *p* = 0.018), cefepime (ICU-1 *p* = 0.028 and ICU-2 *p* = 0.004), meropenem (ICU-1 *p* = 0.009 and ICU-2 *p* = 0.000), ciprofloxacin (ICU-1 *p* = 0.027 and ICU-2 *p* = 0.018) and vancomycin (ICU-1 and ICU-2 *p* = 0.018). However, the use of piperacillin/tazobactam increased in both ICUs. In addition, the incidence of extended-spectrum beta-lactamase (ESBL)-producing *E. coli* and *K. pneumoniae* infections (ICU-1 = 83% and ICU-2 = 78%), also infections with ciprofloxacin-resistant *P. aeruginosa* (ICU-1 = 87% and ICU-2 = 82%) and fourth-generation cephalosporins (ICU-1 83%/ICU-2 76%) were reduced.

Villalobos et al. (2013) implemented a surveillance system in 10 health institutions in Antioquia, Valle del Cauca and Bogotá. The highest consumption in ICUs was of Meropenem antibiotics (MEM); the high resistance of the Enterobacteriaceae family to third-generation cephalosporins (>25.6%), and a high percentage of methicillin-resistant (MR) *Staphylococcus aureus* strains were observed Villalobos [131]. Despite surveillance systems, the scope of these studies is limited, and there is no national record of antibiotic consumption in humans.

As in animals, in humans, antibiotics are not completely metabolised, and certain amounts are excreted into the environment through urine and faeces, contaminating water sources. The principal sources of human antibiotic contamination include waste from pharmaceutical companies, senior residences and hospital wastewaters [132,133,134].

## 5. Presence of Antibiotics in Wastewaters

Traces of antibiotics have long been found in surface, ground and oceanic waters [15,123,135], which has gradually increased reports on the state of contamination, the risks of antibiotics in the environment, the disposal of antibiotics and the prevalence of resistant bacteria from different sectors (aquaculture and livestock, wastewaters and treatment plants) [135,136].

The presence of antibiotics in the environment varies between regions, for example, the oral and parenteral antibiotics that account for at least 75% of total antibiotic consumption in Africa are penicillins (Amoxicillin, AMC), cephalosporins (Ceftriaxone, CRO), quinolones (Ciprofloxacin, CIP), sulphonamides (Sulfamethoxazole, SMX and Trimethoprim, SXT), tetracyclines (Doxycycline, DO), and nitroimidazoles (Metronidazole, MNZ), while in Asia the consumption of cephalosporins and quinolones is prevalent [136,137].

China is considered the world’s largest producer, exporter, and consumer of antibiotics for animal and human use [136]. More than 90 antibiotics belonging to the main classes (sulphonamides, fluoroquinolones, tetracyclines, macrolides, and β-lactams) were monitored from 2005 to 2016; finding concentrations between 0.1 and 1000 ng L^−1^ [137]. Similarly, other studies have reported the presence of these classes of antibiotics in aquatic environments in China. The concentration reported between studies varies from 1 μg L^−1^ to 100 ng L^−1^ [136].

Yang et al. (2004) determined the concentration of macrolides (Erythromycin E, Roxithromycin RXT, and Tylosin TLY) in natural and wastewaters matrices. The concentration (µg L^−1^) of these macrolides in surface water was 93.6 ± 8.6, 92.1 ± 10.0, and 94.3 ± 8.9% for Erythromycin (E), Roxithromycin (RXT), and Tylosin (TLY), respectively. In the influent of a wastewaters treatment plant, the recovery (µg L^−1^) was 84.8 ± 14.0, 83.2 ± 13.1, and 86.1 ± 13.4% for Erythromycin (E), Roxithromycin (RXT) and Tylosin (TLY), respectively [138].

Lien et al. (2016) determined the concentration of antibiotics in wastewaters from an urban hospital in Vietnam before and after treatment over one year. High concentrations of antibiotics, such as Ciprofloxacin (CIP) were found before treatment (171.8 µg L^−1^) and after treatment (71.1 µg L^−1^). Metronidazole (MNZ) was also in high concentrations before and after treatment (328.2 y 133.5 µg L^−1^, respectively). Other antibiotics were detected before and after treatment, such as Sulfamethoxazole (SMX) (87.2 and 26.5 µg L^−1^, respectively) Ofloxacin (OFL) (212.2 and 45.1 µg L^−1^, respectively), Spiramycin (SP) (18.1 and 3.7 µg L^−1^, respectively) and Trimethoprim (SXT) (2.7 and 1.3 µg L^−1^, respectively) [139].

Yang et al. (2004) and Lien et al. (2016) show, respectively, that the load of antimicrobials entering treatment plants could be high and that conventional treatments could reduce this load but they are not biologically significantly.

From 2016 to 2018 in the Americas, the use of some antimicrobials, such as Amoxicillin (AMC), Azithromycin (AZM), Amikacin (AN), Cephalexin (CN), Ceftriaxone (CRO), Cefazolin (CZ), Cefotaxime (CTX), Cefepime (FEP), Ciprofloxacin (CIP), Nitrofurantoin (FM), Sulfamethoxazole (SMX), Trimethoprim (SXT), Dicloxacin (DX), Benzyl-penicillin (PG), Clindamycin (CM), Gentamicin (GM), and Oxacillin (OX), prevailed [21].

In Colombia, the most widely consumed antibiotics by humans are the β-lactams, mainly penicillins, such as Amoxicillin (AMC) (the most consumed by self-medication), Ampicillin (AM) and Dicloxacin (DX), some cephalosporins (Cephalexin CN, Ceftriaxone CRO, and Cephradine RAD), as well as fluoroquinolones (Ciprofloxacin (CIP)). Tetracyclines (Doxycycline DO) and macrolides (Azithromycin AZM) have also been reported [140]. The highest concentration of antibiotics in Colombia has been reported in hospital wastewaters, followed by municipal wastewaters and natural aquatic environments. Antibiotics, such as Ciprofloxacin (CIP), Azithromycin (AZM), and Sulfamethoxazole (SMX) are frequently found in Colombian wastewaters [140].

Additionally, resistant pathogens have been reported worldwide in water and clinical (nosocomial) settings; Gram-negative bacteria, such as *Escherichia coli*, *Pseudomonas aeruginosa*, *Klebsiella pneumoniae*, and *Acinetobacter baumanni* produce β-lactamases and carbapenemases and Gram-positive bacteria, such as methicillin-resistant *Staphylococcus aureus* or vancomycin-resistant *Enterococcus* sp., also produce them [43].

## 6. Global Problem of Antibiotic Resistance

The WHO has recognised antibiotic resistance as a public health threat, as these compounds are increasingly ineffective for infectious disease treatment, and clinical development of new antimicrobials is stagnating. Between 2010 and 2015 the FDA approved eight new antibiotics (Ceftaroline (CPT), Fidaxomicin (FDX), Bedaquiline (BQ), Dalbavancin (DAL), Tedizolid (TZD), Oritavancin (ORI), Ceftolozane-tazobactam, C/T, and Ceftazidime-avibactam, CZA) [141]. In 2018 and 2019, only 9/107 (8.4%) introduced drugs were approved [142]. WHO has stated that new compounds are crucial for infectious disease treatments; however, without better authority, control, and farmers and public responsibility in the use way, the new antibiotics could be ineffective. In our opinion, better control means, in addition to standards, enforcement measures, and sanctions for non-compliance, also includes trying to ensure that antibiotics approved for humans are not approved for animals as far as possible.

On the other hand, approximately a half-million new cases of rifampicin-resistant TB (RR-TB) were detected in 2018; according to the WHO, MDR-TB requires longer treatments, which are less effective and much more expensive than non-resistant TB [143].

According to WHO, the principal causes of antibiotic resistance are misuse and overuse of antibiotics, farms, lack of access to clean water, lack of hygienic conditions in both animals and humans, inadequate measures in health care facilities, limited access to medicines, non-compliance with legislation and lack of knowledge. This situation becomes critical in marginalised, poor, and health crisis regions. Antibiotic resistance rates have increased to the extent that infections caused by resistant pathogens have been treated with last-choice drugs, which may be less effective, less safe, and more expensive; these drugs are often limited and found only in developing countries [43,141].

In 2015, the WHO propitiated a global collaboration to standardise antimicrobial resistance surveillance, the Global Antimicrobial Resistance Surveillance State (GLASS), an organisation involving different countries to produce reliable and comprehensive data, which allowed the assessment of trends in antibacterial resistance between 2015 and 2019. Countries provided data on pathogens isolated from blood, urine, faeces, cervical, and urethral samples. The most frequently reported resistant pathogens were *E. coli*, *K. pneumoniae*, *S. aureus*, *S. pneumoniae*, *Salmonella* spp., *N. gonorrhoeae,* and *Shigella* spp. [43].

In 2017, the WHO published a list of antibiotic-resistant “priority pathogens” to guide and promote research to combat this global problem. It includes 12 genera of bacteria classified as critical, high, and medium priority. Critical priority pathogens included *Acinetobacter baumannii*, *Pseudomonas aeruginosa,* and carbapenem-resistant, ESB-producing Enterobacteriaceae. According to PAHO (Pan American Health Organisation), these microorganisms are dangerous in hospitals, senior residences, nursing homes, and patients using devices, such as ventilators and intravenous catheters. They also cause infections involving blood and lungs, urinary tract, and surgical wounds and can cause nosocomial disease, and are associated with high mortality. The high priority category includes pathogens, such as vancomycin-resistant *Enterococcus faecium*, methicillin- and vancomycin-resistant *Staphylococcus aureus*, clarithromycin-resistant *Helicobacter pylori*, fluoroquinolone-resistant *Salmonella* spp., and cephalosporin-resistant *Neisseria gonorrhoeae*. The medium-priority category includes *Streptococcus pneumoniae* with decreased susceptibility to penicillin, ampicillin-resistant *Haemophilus influenza*, and fluoroquinolone-resistant *Shigella* spp.

## 7. Overviews of Degradation of Antibiotics

Antibiotics are ionisable and can be in the environment as neutral or charged (negative or positive) species. These species have different chemical properties, hence different sorption and degradation mechanisms in soil [144]. Several biotic and abiotic factors influence the degradation rate of these compounds, which is why each can have a different half-life in soil, ranging from <1 to 3466 days [145,146]. Enzymatic hydrolysis is one of the most frequent abiotic degradation pathways. The β-lactams are more susceptible to this degradation than macrolides or sulphonamides, while quinolones and tetracyclines are susceptible to photodegradation. These differences are inevitably dependent on the chemical structure of each class of antibiotics [145].

Several techniques have been assayed for the degradation and mineralisation of antibiotics to reduce the environmental impact [147]; strategies focused on physical, chemical, biological, and combined process design for degradation [147,148,149,150,151,152,153]. Physical and chemical methods include sedimentation, filtration, and oxidation using chemical compounds, UV or Vis light, ultrasound, and ions. Biological ones include the use of microorganisms and or their enzymes [148,149,150].

Unfortunately, many conventional wastewater treatment plants worldwide are not appropriate for removing highly polar micropollutants, such as antibiotics [148]. These plants usually use methods, such as filtration to remove larger solids, whereby wastewaters pass through a granular media, such as sand, charcoal, diatomaceous earth, or activated carbon; subsequently, removing the smaller solids by mechanisms, such as particle retention, electrostatic attraction, or adsorption. These wastes are not degraded and concentrate in a solid phase, generating a new waste. Sometimes, these sludges with antibiotic concentrations ranging from ng kg^−1^ to 100 mg kg^−1^ are used as fertilisers, indicating that the problem has not been solved but shifts the contaminants to other environments [146]. Chemical-assisted sedimentation allows the removal of suspended solids, for which chemical compounds, such as lime (CaO), potassium aluminium sulphate (KAl(SO_4_)_2_ 12H_2_O), or iron salts (FeSO_4_) facilitate the precipitation of solids and their subsequent removal [148]. In addition, conventional wastewaters treatment plants use aerobic or anaerobic processes to remove organic matter [147,149].

Advanced Oxidation Processes (AOP) have been proposed for industrial wastewaters treatments, such as photocatalysis with TiO_2_ using heterogeneous semiconductors, UV photolysis, Photo-Fenton, or electro-Fenton.

Another study used the ferrous ion (Fe(II)-activated decomposition of persulphate (S_2_$O_{2}^{8-}$), for remediation of groundwater containing antibiotics. Hydroxyl (HO^−^) and sulphate (SO_4_^−^) radicals were the main reactive species, and therefore, responsible for the degradation of both Ciprofloxacin (CIP, 95.6%) and Sulfamethoxazole (SMX, 35%) [154].

Chlorinated compounds are frequently used in drinking water treatment plants as disinfectants or oxidising agents. However, the reaction of chlorine with aromatic rings, neutral amines, and double bonds generates halogenated compounds, which may be carcinogenic [147,149].

### 7.1. Biotic Degradation of Antibiotics

Several authors have studied the biodegradation of antibiotics, Jiang et al. (2010) evaluated the degradation of cephalosporins in the surface water and sediments of Lake Xuanwu, China, showing that abiotic hydrolysis (for Cephradine RAD, Cefuroxime CXM, and Cefepime FEP) and direct photolysis (for Ceftriaxone CRO) were the main mechanisms of cephalosporin removal in the surface water of the lake; while aerobic and anaerobic biodegradation removed cephalosporins in the sediment [155].

The degradation of antibiotics in soil depends on physicochemical characteristics [145,146]. Antibiotics accumulate in the soil’s upper layers, which act as a sink; these compounds are washed away by surface water and then leach into groundwater. The transport of antibiotics depends on the chemical properties of each antibiotic and the soil; soil organic carbon content, clay content, texture, pH, and ionic strength can alter the degree of sorption [144,156]. The sorption of antibiotics in the soil can modify their mobility, ecotoxicity, and bioavailability for microbial degradation; with low sorption, there is an increased risk of leaching and increased risk of contamination to groundwater [144,157].

Different classes of antibiotics, such as tetracyclines, sulphonamides, and macrolides, have been quantified in soils, sludge and manure. In sludge, for example, concentrations of Ciprofloxacin (CIP), Norfloxacin (NX) and Tylosin (TLS) have been reported with an average value above 1000 μg kg^−1^. Other antibiotics, such as Tetracycline (TC) and Oxytetracycline (OXT) appeared in sludge with values above 10 μg kg^−1^ dry matter. Additionally, antibiotics in manure have been identified, with concentrations between 100 and 1000 μg kg^−1^ dry matter. The manure or sludge application as fertiliser can facilitate the spread of resistant bacteria in the soil, particularly human pathogenic bacteria. However, their microbial degradation has been also reported [146,156,157,158,159,160,161,162].

Lin and Gan (2011) studied arid soils concerning the capacity of sorption and persistence of Sulfamethoxazole (SMX) and Trimethoprim (SXT). Sulfamethoxazole (SMX) sorption was minimal sorbed concentration (Cs = 0.973 µg kg^−1^), indicating a high risk of leaching, while Trimethoprim (SXT) showed moderate to high sorption (K_d_ = 7.4242 L kg^−1^). The degradation of antibiotics depended on microbial activity, oxygen in the soil, soil type, and antibiotic characteristics [157]. Pan and Chu (2016) obtained similar results; antibiotics belonging to the sulphonamides presented low sorption measured as the adsorption or soil distribution coefficient (K_d_ = 1.365 L kg^−1^), implying a higher risk to groundwater. In contrast, Tetracycline (TE) obtained the highest sorption (K_d_ = 1093 L kg^−1^) value, which would mean a lower risk of groundwater contamination with leachates. Furthermore, the antibiotics evaluated were susceptible to microbial degradation under aerobic and anaerobic conditions. They also showed that, at high antibiotic concentrations, degradation processes are slowest, and their persistence in the soil is prolonged [144].

Microorganisms, such as *Stenotrophomonas maltophilia*, *Sphingobacterium* sp., *Klebsiella* sp., *Bacillus* sp., *Shewanella* sp., and *Trichosporon mycotoxinivorans* participate in microbial degradation of tetracyclines. Several proposed degradation routes are in *Stenotrophomonas maltophilia*; reactions, such as denitromethylation, decarbonylation and deamination have been identified. Likewise, in the degradation route by *Klebsiella* sp., oxidation, hydrolysis ring-opening, decarbonylation, deamination and demethylation reactions are included. Whereas the degradation pathway by the fungus *Trichosporon mycotoxinivorans* includes epimerization, dehydration, and proton-transfer pathway reactions [158].

However, Alexy et al. (2004) evaluated the degradation of antibiotics using the closed bottle technique, which contained a mineral medium, inoculum, the antibiotic, and sodium acetate. Under these conditions, neither Amoxicillin (AMC), Benzylpenicillin (PG), Ceftriaxone (CRO), Cefuroxime (CXM), Chlortetracycline (CTC), Clindamycin (CM), Erythromycin (E), Gentamicin sulphate (GM), Imipenem (IPM), Ofloxacin (OFL) or Sulfamethoxazole (SMX), were biodegraded less than 60% in 28 days. Authors also claim that their removal from the environment by other abiotic mechanisms, such as temperature (thermal treatment) and light (photo treatment) is possible [162].

On the other hand, in a composting process of sludge or manure, antibiotic removal can range from 17 to 100% [146,163]. Composting is one of the techniques used for antibiotic and resistance gene removal in sludge or manure. The persistence and mobility of antibiotics in compost depend on their chemical properties, concentration, and physicochemical conditions, including pH, temperature, total organic carbon (TOC), total nitrogen (TN), total phosphorus (TP), and metal content. However, the removal efficiency of residual antibiotics, and antibiotic resistance genes, is still very low [163].

### 7.2. In Situ Chemical Oxidation (ISCO) of Antibiotics

In situ chemical oxidation is a removal strategy for different compounds, based on the addition of oxidising chemical compounds into the soil and groundwater to remove organic pollutants. Several authors have studied the degradation of antibiotics based on this strategy: thermo-activated persulfate oxidation [164], peroxymonosulfate oxidation [165], ozone oxidation [166], or ozone-based advanced oxidation process [167]. Wu et al. (2020) used a peroxymonosulfate activation support (amorphous CoSx cages advanced oxidation catalysts) to detect the degradation of Tetracycline (TE, 90%) and Ciprofloxacin (CIP, 90%). They also identified the intermediates and possible degradation routes after peroxymonosulphate activation, in which reactive species (SO_4_, O_2_, and OH) formed, and oxidised Tetracycline (TE) in reactions, such as deamidation, hydroxylation and the ring-opening reaction. They suggested that some intermediates (SO_4_·^−^/O_2_·^−^/·OH) could be mineralised (H_2_O and CO_2_) [168].

Guo et al. (2013) used Co_3_O_4_-catalyzed peroxymonosulfate oxidation based on sulphate radicals for Amoxicillin (AMC) degradation. This process is based on the generation of sulphate radicals through Co_3_O_4_-mediated peroxymonosulphate (PMS) activation. The authors observed a decrease in Chemical Oxygen Demand (COD) of 91.01% [169].

### 7.3. Photocatalytic Advanced Oxidation Processes (AOP) for Heterogeneous Degradation of Antibiotics

AOP are advanced oxidative processes based on the generation of highly reactive hydroxyl (OH*) radicals. These radicals come from oxidising agents, such as hydrogen peroxide (H_2_O_2_) or ozone (O_3_), usually using catalysts, such as titanium dioxide (TiO_2_) and UV light, which is known as heterogeneous catalysis [170]. Several authors reported AOP removal rates (>90%) for penicillins, sulphonamides, phenicols, β-lactams, tetracyclines, and fluoroquinolones [171,172,173,174,175]. However, the toxicity may not be totally reduced, and some intermediates generated could be more toxic than the original compounds [147,176]. On the other hand, AOPs combined with biological treatments (gamma irradiation, ionising irradiation integrated with activated sludge, a biological treatment combined with flocculation and ultrafiltration) have shown high removal yields (close to 100%) of toxicants and recalcitrants [174,177].

#### 7.3.1. Photodegradation of Antibiotics Using TiO_2_ Based Heterogeneous Semiconductors

Photocatalysis using semiconductors is an indirect photocatalytic AOP that uses TiO_2_ electrodes for organic and inorganic compound oxidation–reduction reactions. TiO_2_ is non-toxic and has a high catalytic activity; however, its catalytic activity is attenuated by solar irradiation; therefore, other irradiation techniques, such as ultraviolet light (UV) have been applied [170,178].

Some authors have reported antibiotic removal rates higher than 80%, under certain specific conditions (radiation intensity, composition, concentration of the hybrid material, the matrix used, pH and temperature), in the degradation of Ciprofloxacin (CIP, 88%), Gatifloxacin (GAT, 96%), Levofloxacin (LVX, 96%), Tetracycline (TE, 83%), Penicillin (P, 90%), Sulphonamide (SUL, 97%), Sulfamethoxazole (SMX, 80% mineralisation), Ampicillin (AM, 98%) and Erythromycin (E, 90%) [170]. Others have evaluated the degradation of penicillins (Amoxicillin AMC, Ampicillin AM, and Cloxacillin CLO) by photocatalysis with TiO_2_ and H_2_O_2_, finding 100% degradation [179].

Degradation of Amoxicillin (AMC), Ampicillin (AM), and Cloxacillin (CLO) using ZnO photocatalysis reported rates above 80% [180]. Zheng et al. (2018) utilised a graphitised mesoporous carbon (GMC)-TiO_2_ nanocomposite, adsorbing the antibiotics efficiently. Subsequently, the photocatalysts and reactive species (hydroxyl radical) were contacted for antibiotics degradation; 15 mg L^−1^ of Ciprofloxacin (CIP) was fully mineralised (CO_2_ and H_2_O) in 1.5 h [181].

Abellán et al. (2009) by using several concentrations of TiO_2,_ assayed Sulfamethoxazole (SMX) and Trimethoprim (TMP) removal; degradation >90% was observed for both compounds; optimal concentrations were 1.0 and 0.5 g TiO_2_ L^−1^, respectively. They also observed that SMX reduced its aromatic content, while TMP increased its aromatic content during the first three hours due to the formation of dimers; finally, the aromatic content decreased [182].

#### 7.3.2. Photodegradation of Antibiotics Using Non-TiO_2_ Based Semiconducting Catalysts

Other catalysts than TiO_2_ have been used for antibiotics removal; for example, the hybrid material composed of co-modified NiS and MoS_2_ nanoflakes (g-C_3_N_4_-NiS-MoS_2_), synthesised by the direct mixing of graphitic carbon nitride with nickel salts and thiourea, has also used. Tetracyclines have been the most studied compounds using this catalyst, obtaining degradation rates >90% [170]. Several authors have evaluated different hybrid materials as catalysts for antibiotic degradati4n and have shown them to be efficient systems; using the Ppy-BiOI catalyst, 54% and 61% degradation was obtained for Chlortetracycline (CTC) and Tetracycline (TE), respectively. The hybrid material CQDs-Bi_2_WO_6_ degraded 87% of Ciprofloxacin (CIP) in 120 min., [178,183,184,185,186,187,188,189].

#### 7.3.3. Photo-Fenton or Electro-Fenton of Antibiotics Removal

Fenton is an advanced oxidation process in which chain reactions mediated by a non-toxic catalyst (Fe^2+^), an acidic medium (pH = 2.8–3.0), and an oxidant (H_2_O_2_) take place for the formation of hydroxyl radicals (OH). Several studies address the degradation of Tetracyclines (TE), Norfloxacin (NX), and Penicillin (P) using this method, showing degradation rates >80% [170,190]. Likewise, the large-scale practical application of the Fenton process has disadvantages due to the large amount of ferric sludge it generates. This solid waste sludge is considered a potential hazard due to the adsorbed organic residues of the treated wastewaters. Therefore, special treatment is required, and solid waste disposal must be at specific locations [158].

### 7.4. Degradation of Antibiotics in Water by Plasma Treatment

Another technique used for antibiotic removal is the dielectric barrier plasma discharge (DBD); this technique generates oxidising species: radicals (H, O, OH) and molecules, such as H_2_O_2_ and O_3_ for the removal of pollutants in the air and water [190]. Plasma treatment has a strong oxidising capacity, high efficiency, and experimental ease and does not require exogenous reagents. Magureanu et al. (2011) evaluated the degradation of Amoxicillin (AMC, 100%), Oxacillin (OX, 100%), and Ampicillin (AM, 100%). Amoxicillin (AMC) was degraded after 10 min of dielectric barrier discharge plasma, while the other two antibiotics degradation took around 20 to 30 min [191].

Kim et al. (2013) showed the degradation of Lincomycin (LIN), Ciprofloxacin (CIP), Enrofloxacin (ENR), Chlortetracycline (CTC), Oxytetracycline (OXT), Sulfathiazole (STZ), Sulfamethoxazole (SMX), and Trimethoprim (SXT). The results indicated that in degradation by dielectric barrier discharge plasma, the degradation rates were >90% but depending on the amount of energy supplied, and each antibiotic showed a different degradability [192].

Li et al. (2020) also assayed Tetracycline (TC), Sulfadiazine (SD), and ciprofloxacin (CIP) degradation by non-thermal discharge plasma. All antibiotics showed different degradation rates, and 20 min after plasma treatment at 19 Kv, found degradation efficiencies of 93.3, 81.2, and 58.5% for TC, SD, and CIP, respectively. The clearance efficiency and mineralisation yield of Tetracycline (TE) were relatively higher than the other antibiotics [190].

Sarangapani et al. (2019) evaluated the degradation of Ofloxacin (OFX) and Ciprofloxacin (CFX) by the atmospheric cold plasma method. Such plasmas generate the chemical reactions responsible for the formation of reactive species. The degradation was 92% and 89% for OFX and CFX, respectively, reducing the activity of both antibiotics [193].

### 7.5. Cathodic Degradation of Antibiotics

Cathodic degradation is an emerging technology that has potential in wastewaters treatment for nitro compounds, aromatics, azo dyes and halogenated compounds removal. This technique provides electrons for reductive degradation of pollutants, is environmentally friendly as no chemicals are needed, requires low voltage application and is low-cost [194]. Kong et al. (2015) assayed the degradation of Nitrofurazone (NFZ, 98.71%), Metronidazole (MNZ, 98.21%), Chloramphenicol (CHL, 99.86%) and Florfenicol (FLO, 99.72%) by using a dual-chamber electrochemical reactor. The different cathodic potentials applied generated a high efficiency in the degradation of the antibiotics. Finally, the authors conclude that electrochemical reduction is promising as a pretreatment or advanced treatment of wastewaters containing antibiotics [194].

### 7.6. Temperature Degradation of Antibiotics

Several authors have studied the effect of different temperatures on antibiotic degradation. Lin et al. (2017) evaluated the temperature effect on the degradation of antibiotics in manure (one of the main reservoirs of antibiotics and resistant bacteria); they incubated pig and chicken manure at 30, 40, 50 and 60 °C for five days, identifying the presence of different classes of sulphonamides. In pig manure, they found Sulfadiazine (SDZ), Sulfadimethoxine (SDM), Sulfamethoxine (SMZ) and Sulfamethamonomethoxine (SMM); in chicken manure, they found Sulfadiazine (SDZ), Sulfadimethoxine (SDM), Sulfamethoxine (SMZ) and Sulfaquinolaxine (SQ). The temperature effect varies depending on manure origin and antibiotic class; at 60 °C, the residual concentration of antibiotics in pig manure decreased up to 2 mg kg^−1^. In contrast, the sulphonamides in chicken manure were the lowest at 30 °C (0.5 mg Kg^−1^); however, they increased at 40 °C (7 mg Kg^−1^) and then decreased continuously as the temperature increased [195].

Loftin et al. (2008) tested the degradation at different temperatures (7, 22, and 35 °C), pH (2, 5, 7, 9, and 11), and ionic strength (0.0015, 0.050, and 0.084 mg L^−1^) of Chlortetracycline (CTC), Oxytetracycline (OXT), Tetracycline (TE), Lincomycin (LIN), Sulfachloropyridazine (SCP), Sulfadimethoxine (SDM), Sulphathiazole (STZ), Trimethoprim (SXT), and Tylosin (TYL), observing that temperature and pH affected the degradation rate of CTC (22 °C, pH 9 ± 0.2), OXT (22 °C, pH 7 ± 0.2), and TE (35 °C, pH 9 ± 0.2), which increased with increasing temperature [150].

### 7.7. Sonocatalytic Degradation of Antibiotics

Sonocatalysis is a recently advanced oxidation process (AOPs) used for water treatment using ultrasound; this process induces acoustic cavitation “in situ” to remove contaminants in a liquid phase [196,197].

Cavitation is the formation, growth and implosive collapse of gas- or vapour-filled microbubbles (considered microreactors) resulting from the acoustic wave/rarefaction-induced compression in a liquid [197]. Due to the high temperatures (≥5000 K) and pressures (≥1000 atm) reached during the process, an oxidative environment is generated with highly reactive species (ROS), such as hydrogen free radicals H*, hydroxyl (*OH) and hydroperoxyl (HO_2_*) [196,197]; species capable of degrading recalcitrant contaminants, attacking organic molecules instantaneously [196].

De Bel et al. (2009) used sonolysis at 520 kHz and 92 WL^−1^ for the degradation of Ciprofloxacin (CIP), finding that the degradation rate is pH-dependent due to the degree of protonation and positive charges acquired by Ciprofloxacin (CIP) during treatment which favour ultrasonic degradation. In this study, the degradation rate increased, since the pseudo-first-order degradation constant increased almost four times when comparing the treatment at pH 3 (0.021 min^−1^), pH 7 (0.0058 min^−1^) and pH 10 (0.0069 min^−1^), obtaining a BOD/COD ratio of 0.06 to 0.60; 0.17 and 0.18, after 120 min of irradiation, respectively [198].

Sonochemical degradation of antibiotics has been promisingly successful due to TOC (Total Organic Carbon) reduction, COD (Chemical Oxygen Demand) removal and an increase in BOD_5_ (Biological Oxygen Demand) and BOD_5_/COD ratio. In this regard, Liu et al. (2021) provide an overview related to the removal efficiency (RE) of various antibiotics using different conditions, such as ultrasonic frequency (US, 20–600 kHz); electrical power input (P_E_, 60–860 W); sonication times (t, 20–300 min) by using removal processes, such as Sonication alone, Sonocatalysis, Sono/Fenton, Sono/PS, Sono/Photo, and Sonozonation. In general, summarized results by Liu et al. (2021) showed different removal efficiencies for antibiotics, such as Sulfamethazine (SM2), Oxacillin (OX), and Dicloxacillin (DCX), all of them with 100% RE; Sulfadiazine (SD, 90% RE); Cephalexin (CN), Ciprofloxacin (CIP), Penicillin G (PG), all of them with 52–66. 7% RE; Ofloxacin (OFL, 31% RE); Levofloxacin (LVX, <10% RE) [199].

However, the results reviewed by Liu et al. (2021) also showed that: (i) the antibiotic’s distance from cavitation bubbles is crucial, (ii) that removal efficiency (RE) is pH-dependent in some cases, (iii) that the hydrophobicity of the antibiotic is critical for some processes, (iv) that in some cases mineralization is not achieved, and (v) that molar volume is critical for reactions [199]; indicating that even some adjustments are necessary for the treatment of each molecule; however, the residuals to be treated are a mixture of these target molecules, among others.

**Figure 1:**
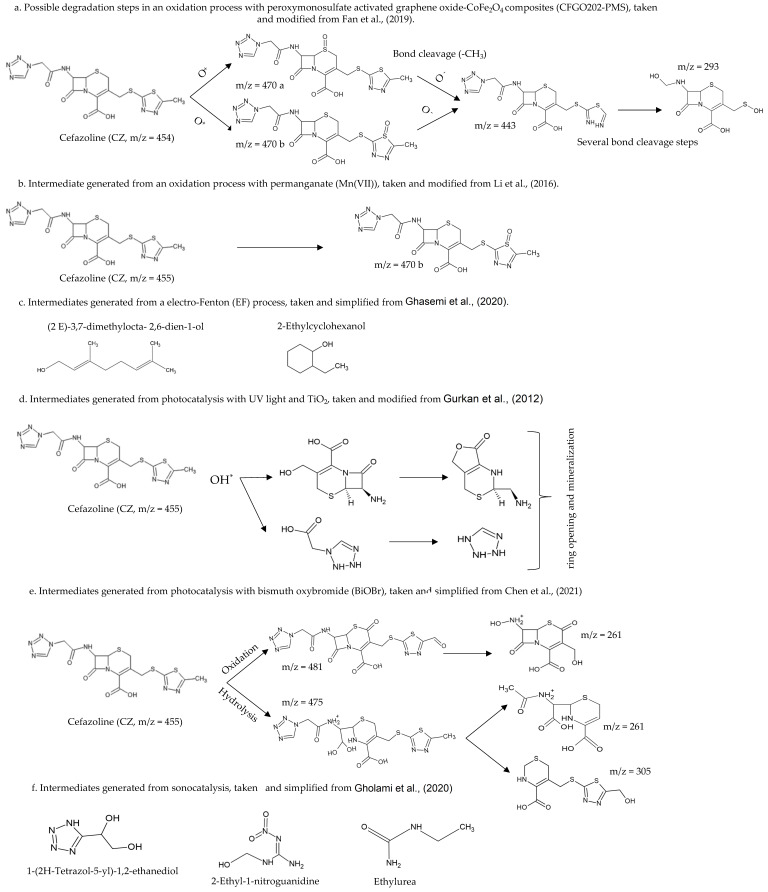
Some intermediates generated and degradation steps of Cefazolin (CZ) from different techniques, *m*/*z* is the mass to charge ratio. (**a**) degradation of Cefazolin (CZ) in an oxidation process with activated peroxymonosulfate (PMS) [202]. (**b**) intermediates generated from an oxidation process with permanganate (Mn (VII)) [200]. (**c**) Intermediates generated from a Fenton process [203]. (**d**) Intermediates generated from photocatalysis with UV light and TiO_2_ [204]. (**e**) Intermediates generated from photocatalysis with bismuth oxybromide (BiOBr) [205]. (**f**) Intermediates generated from sonocatalysis [206].

In summary, the biotic, physical and chemical antibiotic degradation processes have obtained high elimination percentages (72.5–90%); however, only a few authors have reported the intermediates and possible degradation steps or sequence, because intermediates compounds generated vary according to the antibiotic degraded, the process (biological, chemical, physical, or combined) and the conditions used (pH, temperature or presence of chemicals) [200]. The identification of intermediate compounds resulting from antibiotic degradation, their toxicity, and residual antimicrobial activity is crucial to establishing the process efficiency; likewise, knowing the different degradation steps is the key to improving each treatment [201]. In this respect, Fan et al. (2019) proposed a degradation route of Cefazolin (CZ) from an advanced oxidation process with activated peroxymonosulfate (PMS); high molecular weight sulfoxide compounds were identified (Figure 1a) [202]. Some authors also have identified intermediates generated in the degradation of Cefazolin (CZ) by using oxidation reactions; Li et al. (2016) used permanganate (Mn (VII)) as an oxidant and identified five intermediates, of which one of these matches the compounds detected by Fan et al. (2019), (Figure 1b) [200]. Ghasemi et al. (2020) determined the intermediate compounds generated from the degradation of Cefazolin (CZ) from an electro Fenton (EF) process, identifying low molecular weight compounds, so the authors claim that their mineralisation using a biological treatment is possible (Figure 1c) [203].

On the other hand, the possible Cefazolin (CZ) degradation after photocatalysis processes have proposed; Gurkan et al. (2012) used UV light and TiO_2_, and the authors determined that the degradation of Cefazolin (CZ) occurs through intramolecular cleavage of lactam rings, thiadiazole, tetrazole, and subsequent reactions with -OH* radicals that transform the fragments into smaller species [204]. Figure 1d shows some of the intermediates generated. Chen et al. (2021) used a bismuth oxybromide (BiOBr) photocatalyst for the removal of Cefazolin (CZ) [205]; in this study, hydrolysis and oxidation reactions were identified (Figure 1e). Other techniques, such as sonocatalysis have also identified some intermediates (Figure 1f) [206].

**Figure 2:**
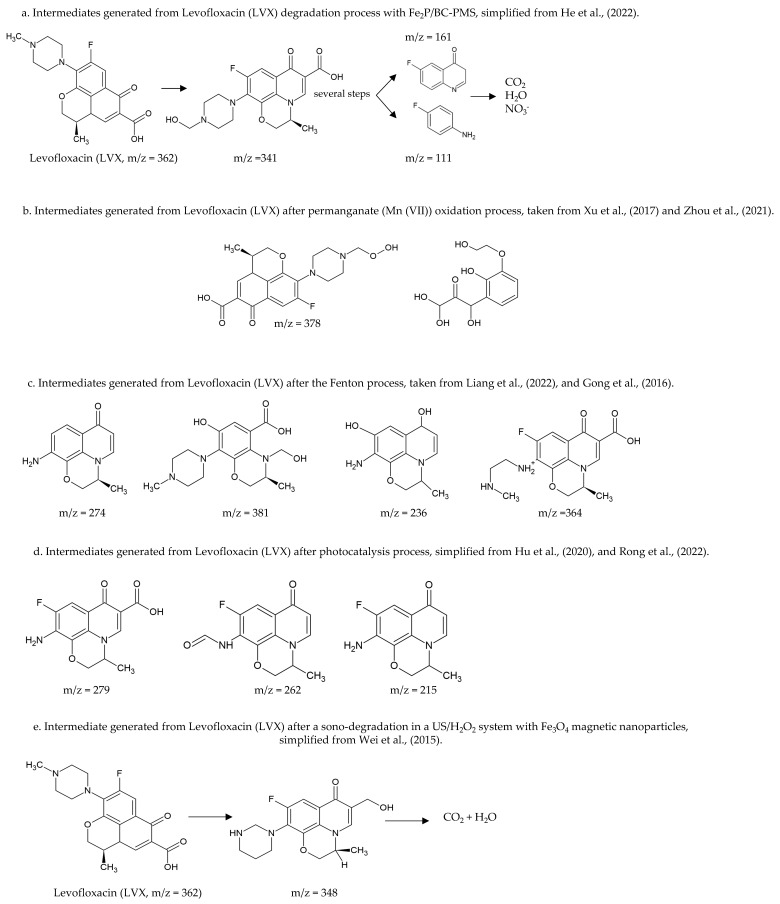
Some intermediates and degradation steps of Levofloxacin (LVX) by employing different techniques for degradation, *m*/*z* is the mass to charge ratio. (**a**) degradation pathway of Levofloxacin (LVX) in an oxidation process with peroxymonosulfate (PMS) [207,208]. (**b**) Intermediates generated from an oxidation process with permanganate (Mn (VII)) [209,210]. (**c**) Intermediates generated from a Fenton process [211,212,213,214]. (**d**) Intermediates generated from photocatalysis [202,215,216]. (**e**) Intermediates generated from sonocatalysis [217].

Additionally, Levofloxacin degradation (LVX) has been extensively studied by different methods. Liu et al. (2021) and He et al. (2022) identified the intermediates generated from chemical oxidation using peroxymonosulfate; these authors, despite studying the degradation of the same molecule using the same technique, have found that the conditions surrounding the reactions crucially influence the generated intermediates to be different; however, both authors reported obtaining small molecules that can be mineralized [207,208]. Figure 2a shows some intermediates found [207,208]. Other studies have reported the degradation of Levofloxacin (LVX) by chemical oxidation using permanganate (Mn (VII)) (Figure 2b) [209,210]. Additionally, other authors evaluated the degradation of Levofloxacin (LVX) using Fenton and proposed the possible degradation steps (Figure 2c) [211,212,213,214].

Moreover, other authors have evaluated the degradation of Levofloxacin (LVX) using photocatalysis under different conditions, generating different compounds (Figure 2d) [202,215,216]. Sonocatalysis also has been tested for the degradation of Levofloxacin (LVX); Wei et al. (2015) proposed the possible degradation steps to achieve the mineralization of the compound (Figure 2e) [217].

Table 2 summarises some biotic, chemical, physical and combined treatments for the degradation of various antibiotics.

## 8. Enzymatic Degradation of Antibiotics

For several years, the biodegradation of antibiotics by microorganisms, such as bacteria and fungi has been carried out. For example, aerobic heterotrophic bacteria, such as Actinobacteria or Proteobacteria can transform sulphonamides (to N_4_-acetylsulfamethoxazole) or even mineralise them (CO_2_ and H_2_O). However, in some antibiotics, only minor changes of the molecule occur without changing the activity, while for others, intermediaries are transformed back to the parent form; as occurs in the transformation of sulphonamides to N_4_-acetylsulfamethoxazole, which can be reversible [228]. In addition, the ability to degrade antibiotics is limited in some bacteria by environmental conditions [228].

Some techniques use enzymatic degradation under controlled conditions for wastewater treatment, such as the activated sludge bioreactor (ASB), in which pollutants are submitted to aeration to promote the growth of bacteria that degrade toxic organic substances [229]. The ASB is the most widely adopted system for biological wastewater treatment; however, this method is not for antibiotics removal; for which the concentration of antibiotics removed can vary between classes [230].

On the other hand, the use of membrane bioreactors (MBR) is one of the alternatives for the removal of antibiotics, such as Ciprofloxacin (CIP), Erythromycin (E), Tetracycline (TE), Ofloxacin (OFL) and Chlortetracycline (CTC) with removal rates higher than 90% [231]. In MBR, the mechanism for antibiotics removal is enzymatic biodegradation, which is a promising alternative to eliminating these contaminants due to its low energy requirements and high efficiency [232].

Oxidase enzymes have been the most studied enzymes in the degradation of antibiotics. Ligninolytic enzymes produced by fungi, bacteria, plants and algae, including laccases (Lac, E.C. 1.10.3.2), lignin peroxidase (LiP, E.C. 1.11.1.14), manganese peroxidase (MnP, E.C. 1.11.1.13), and versatile peroxidase (VP, E.C. 1.11.1.16), have been assayed for antibiotic removal, showing high degradation rates 72.5–90%) [4,233,234,235].

For example, Wen et al. (2019) evaluated the degradation of Oxytetracycline (OXT) by immobilising LiP enzymes from *Phanerochaete chrysosporium* on bentonite-derived mesoporous materials; the degradation was 95% [236]. Several authors have identified the intermediates generated in biotransformation, their toxicity and possible degradation routes. Tian et al. (2020) evaluated the degradation of Oxytetracycline (OXT) using laccase produced by the fungus *Pycnoporus* sp., in the presence of ABTS and Al^3+^, Cu^2+^, and Fe^3+^ ions. Authors showed that OXT was 100% degraded, and the antimicrobial activity reduced after treatment. In this work, the degradation pathway includes the reactions of deamination, demethylation, and dehydration. Furthermore, seven intermediates in the degradation of OXT were identified [237].

Copete-Pertuz et al. (2018) evaluated the degradation of Oxacillin (OX), Cloxacillin (CLX) and Dicloxacillin (DCX) using the crude extract produced by the fungus *Leptosphaerulina* sp. The three antibiotics were 100% degraded, and laccase (Lac, E.C. 1.10.3.2) and versatile peroxidase (VP, E.C. 1.11.1.16) were the responsible enzymes for antibiotics transformation. The authors also demonstrated the lost antimicrobial activity of the three antibiotics and that intermediaries were shown to be non-toxic by the cytotoxicity assay [238].

Zhang et al. (2020) evaluated the degradation of antibiotics, such as Ampicillin (AM) and Tetracycline (TE) by immobilising the laccases produced by the bacterium *Bacillus subtilis* in a copper-trimic acid (Cu-BTC) framework. Without chemical mediators in the enzymatic treatment, a high degradation efficiency for both antibiotics (close to 100%) resulted. Eleven and fourteen intermediates, respectively, from Tetracycline (TE) and Ampicillin (AM) degradation, and the possible degradation routes, were identified. In addition, loss of antimicrobial activity and low ecotoxicity of the degradation products generated was demonstrated [67].

Sun et al. (2021) evaluated the degradation of tetracycline (TE) using the MnP enzyme. Eighty percent of Tetracycline (TE) was degraded within three hours, showing the antimicrobial activity of the transformation products from Tetracycline (TE) decreased over the reaction time. Seven compounds resulted from TE degradation and proposed a possible degradation route (demethylation, dimethylamino oxidation, decarbonylation, hydroxylation, and oxidative dehydrogenation) Sun [239].

In contrast, other authors have demonstrated the formation of toxic products due to the presence of chemical mediators. The effect of a mediator on oxidation depends on the laccase type, the substrate, the radicals formed, the mediator recyclability, and the laccase stability [4].

Weng et al. (2012) reported the degradation of Sulphadimethoxin (SDM) and Sulphamonomethoxin (SMM) using violuric acid (VLA), syringaldehyde (SIR), and 4-hydroxy benzyl alcohol (HBA) as mediators of enzymatic activity. Degradation of antibiotics using laccase in the presence of the mediators VLA or HBA generated degradation products of lower toxicity, in contrast to the treatments where the mediators were ABTS or SIR, which showed high toxicity [240].

Becker et al. (2016) reported that the mediator ABTS increased the degradation efficiencies of Sulfamethoxazole (SMX), Ampicillin (AM), and Trimethoprim (SXT), but decreased the degradation efficiency of Tetracycline (TE) and Oxytetracycline (OXT), furthermore, laccase was not able to transform Chloramphenicol (C) in the presence or absence of ABTS. The laccase combined with ABTS treatment, induced non-specific toxicity in some bioassays, involving the generation of transformation products or toxic radicals [4].

In some studies of antibiotic biotransformation, the degradation pathways, the intermediates formed, and the microorganisms and enzymes involved at each stage are still unclear. There are antibiotics widely used in humans and animals; however, their enzymatic degradation has not been assayed [231].

Chemical techniques for the removal of contaminating compounds have shown high efficiency in the degradation of antibiotics; however, they have high costs. In most of them, pollutant mineralisation is flawed, while more toxic degradation intermediates remain accumulated. For this reason, several authors have proposed that combined degradation and biodegradation processes may be the best solution for wastewater treatment. The enzymatic treatment can be pre- or post-chemical-treatment in conventional wastewaters treatment plants (WWTPs) to increase biodegradability or degrade recalcitrant compounds that were not completely removed or mineralised at the conventional ones [231]. A higher removal rate of about 20 to 50% of hybrid processes appears to be more effective for recalcitrant antibiotics removal [229].

### 8.1. Laccases

Laccases (Lac, E.C. 1.10.3.2) are enzymes belonging to the cupredoxin superfamily, specifically the multi-copper blue oxidase family [241]. They are ubiquitous, found in fungi, bacteria, plants, and insects, organisms in which they play different roles. In fungi, laccases allow the depolymerisation of complex compounds, such as lignin [242] morphogenesis, pigment production, and defence against stress [243]. Different genera producing laccases have been studied, such as white-rot fungi [244]. They are the most efficient organisms in lignin degradation until their mineralisation to CO_2_ and water. Lignin is the second most abundant biopolymer after cellulose and is one of the most recalcitrant natural compounds [245]. Lignin degradation, like that of other xenobiotic compounds, occurs due to the production of low specificity extracellular ligninolytic oxidoreductase enzymes, classified as phenol oxidases and haem-peroxidases [246]. Phenol-oxidase enzymes use oxygen (O_2_) as the final electron acceptor, whereas haem-peroxidases use hydrogen peroxide (H_2_O_2_) as the final electron acceptor. Phenol-oxidase enzymes include laccases, while haem-peroxidases include enzymes, such as lignin peroxidase (LiP, E.C 1.11.1.14), manganese peroxidase (MnP, E.C 1.11.1.13), versatile peroxidase (VP, E.C 1.11.1.16) and dyP-type peroxidases (E.C. 1.11.1.19) [242,247,248]. Additionally, there are accessory enzymes involved in lignin degradation, which produce hydrogen peroxide (H_2_O_2_) required by peroxidases, such as aryl alcohol oxidase (E.C. 1.1.3.7), glyoxal oxidase (E.C. 1.2.3.5) and glucose 1-oxidase (E.C. 1.1.3.4) [246]. The activity and ability of these enzymes to degrade compounds vary according to species and catalytic properties [248]. Laccase enzymes are the main enzymes involved in lignin degradation, and the rate of lignin degradation is related to the production of laccases. Currently, laccases are of great industrial importance as they can catalyse the oxidation of a large number of compounds and are also of great importance in the treatment of pesticides, explosives, wastewaters and synthetic dyes [243], and wastes generated by different industries, mainly paper, petrochemical and textile industries [249]. These waste compounds harm the environment; for example, synthetic dyes have complex chemical structures that are difficult to degrade and harm the environment and health, as they are toxic, carcinogenic and highly recalcitrant [250,251,252].

#### 8.1.1. Laccase and the Degradation of Antibiotics

##### Laboratory Studies

Tetracycline’s broad action spectrum and low-cost favours its wide use in humans and animals. These antibiotics are frequently in wastewaters, and their degradation contributes to a significant decrease in antibiotic contamination. Therefore, Tetracycline (TE) degradation by using laccases has previously been reported. Zhang et al. (2020) obtained a degradation percentage of tetracycline (1 mg mL^−1^) close to 100%, using immobilised laccases from *Bacillus subtilis* [67].

Yang et al. (2017) immobilised laccases from *Cerrena unicolor* to assay the degradation of six antibiotics, Oxytetracycline (OXT), Tetracycline (TE), Trimethoprim-Sulfamethoxazole (SMX), Ampicillin (AM), Erythromycin (E), and Chloramphenicol (CHL), with or without ABTS as a mediator of enzyme reaction. The highest degradation (80%) was found on Tetracycline (TE) (100 µg mL^−1^), using 40 U mL^−1^ of laccases, pH 6.0 ± 0.2, 25 °C in 12 h [253].

Becker et al. (2016) evaluated the degradation of 38 antibiotics of different classes in an enzymatic membrane bioreactor by immobilising laccases produced by *Trametes versicolor*. In the presence of the mediator syringaldehyde, the degradation of 32 antibiotics (10 µg L^−1^) was greater than 50% in 24 h [4]. Similarly, Navada et al. (2019) evaluated the degradation of Chloramphenicol (CHL) (10 mg mL^−1^), a recalcitrant and thermostable antibiotic, using laccases produced by *Trametes hirsuta* in the presence of the mediator ABTS (0.25 mM) and observed 82% degradation in 48 h [254].

**Figure 3:**
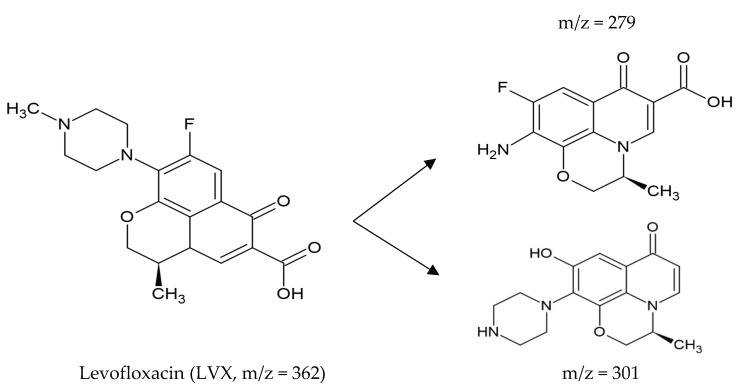
Intermediates resulting from the degradation of Levofloxacin (LVX) using an osmotolerant laccase (E.C. 1.11.1.7) from *Trametes versicolor* in the presence of urea, *m*/*z* is the mass to charge ratio. Taken and simplified from [255].

Najafabadipour et al. (2021) proposed degradation of Levofloxacin (LVX), generated from an enzymatic reaction with an osmotically stable laccase (E.C. 1.11.1.7) in a urea-containing solution, (Figure 3).

Moreover, some drugs are cytotoxic, mutagenic, teratogenic and carcinogenic, even at low concentrations; doxorubicin has been detected in hospital wastewaters, as it is excreted in urine from 3 to 10% and in faeces from 40 to 50% of the dose administered to the patient. Doxorubicin at concentrations of 0.05 μg L^−1^ causes DNA damage in *Ceriodaphnia dubia*. Kelbert et al. (2020) evaluated the degradation of doxorubicin using laccases from *Trametes versicolor*. The laccases degraded different concentrations (50, 250, and 500 μg L^−1^) of doxorubicin in 4 h; the assay conditions were 1800 UL^−1^ of laccases, pH 7.0 ± 0.2, and 30 °C [256].

##### Computational Studies for Antimicrobial Degradation Using Laccases

Bioinformatics tools facilitate the prediction of molecular mechanisms and the behaviour of enzymes against different ligands to obtain a prior estimation of their industrial potential use in bioremediation processes. Moreover, it is economically convenient to perform this computational simulation before experimental trials. The interaction enzyme-substrate can be studied through molecular docking [257,258,259,260,261,262,263] and molecular dynamics analyses [264]; for these studies, algorithms allow for calculating the electrostatic interactions of the amino acid residues in contact with the specific substrates (ligands) to be studied [265].

Computer tools allow the prediction of the properties of a molecule by homology modelling of its 3D structure. Structural knowledge of molecules is a prerequisite for molecular docking analyses, but this is the major limitation because, in most cases, the 3D structure of proteins is unknown, as they have not been purified and crystallised [257,259,260,261,262,263,264,266]; making it necessary to work with simulated models.

Experimental procedures for structure determination have certain disadvantages and limitations because, during the crystallisation of highly hydrated crystals, they can distort some regions as it is sometimes unclear whether an atom corresponds to the protein or the water molecule. Likewise, using techniques, such as Nuclear Magnetic Resonance (NMR) can determine the structure of proteins; however, these must be small proteins (less than 30 kDa), and difficult to crystallise [260]. Experimental structure determination is also expensive and time-consuming [267].

There are computational studies on interactions between laccases and different pollutants from the pharmaceutical industry. Singh et al. (2015) evaluated the interaction of laccases from *Trametes versicolor* with several pollutants from the pharmaceutical industry (Roxithromycin (RXT), Clarithromycin (CLR), Indomethacin (IMT), Bezafibrate (BZ), Metoprolol (MTL), Celiprolol (CPL), and Iopromide (IPD)), using ABTS as docking control molecule (−328.4 Kcal mol^−1^). Roxithromycin (RXT), Clarithromycin (CLR) and Iopromide (IPD) obtained lower energy values concerning the ABTS (−396.7, −380.5, −337. 7 Kcal mol^−1^, respectively), concluding that these contaminants are susceptible to degradation by laccases, contrary to the rest of contaminants that obtained higher energy values than ABTS, such as Indomethacin (IMT) (−294.0 Kcal mol^−1^), Bezafibrate (BZ) (−304.7 Kcal mol^−1^), Metoprolol (MTL) (−263.6 Kcal mol^−1^) and Celipropol (CPL) (−299.7 Kcal mol^−1^) [268].

Sakar et al. (2020) analysed the computational structure of laccases produced by *Thermus thermophilus*; the enzymes were characterised under physicochemical parameters, determining that they are mainly composed of negatively charged aliphatic amino acids, mostly valine and to a lesser extent cysteine. Additionally, due to the high value of the aliphatic index (41.29) and the presence of salt bridges in their structure, they are thermostable enzymes. In addition, identified random folding regions played a role in the flexibility and conformational changes of the protein. On the other hand, the Ramachandran analysis shows the statistical distribution of the torsion angles of the amino acid residues of a protein in the sterically favoured, allowed and not allowed regions; in this case, more than 90% of the amino acids were located in protein regions that were energetically favoured; demonstrating a high quality of the model. In addition, according to the negative value of hydropathy, they confirmed the hydrophilic nature of laccases and identified the binding sites and functional motifs [267].

Cárdenas-Moreno et al. (2019) computationally evaluated the interactions of *Ganoderma weberianum* laccases with different pharmaceutical compounds, including Tetracycline (TE) and Sulfixazole (SXZ). The authors modelled and optimised the laccase 3D structure, identifying the active substrate binding sites, and the allowed angular conformations to establish the docking coordinates and modelled the ligands. The authors ran analyses taking into account random conformations and the number of interactions, using ABTS (−7.1 kcal mol^−1^) and DMP (−4.8 kcal mol^−1^) as controls. Tetracycline (TE) has no interaction with any binding site; however, it had an energy of −5.2 kcal mol^−1^, probably due to the high density of polar groups unable to interact with the amino acid residues of the enzyme active site. Sulfisoxazole (SXZ) had an energy of −6.8 kcal mol^−1^; a hydrophobic interaction with the His^423^, which is in the H-X-H motif at the CuIII copper-binding site, and a minor atomic interaction distance of 7 Å, was detected. This distance between a ligand atom and a receptor atom indicates the relative orientation of the ligands. Small distances (<1.5 Å) represent an atomic overlap generating a major repulsive force, while large distances result in a less effective energy constant [269].

On the other hand, the authors calculated the root mean square deviation (RMSD), in this type of analysis, the structural comparison results from the alignment with crystallised structures. The lower the RMSD is, the higher the similarity between the compared molecules. However, this criterion depends on molecule size [260,270] because values lower than 3.0 Å (typical RMSD values for homologous proteins) indicate high-quality models. In this case, an RMSD = 1.991 was obtained for laccase-Sulfixazole and RMSD = 1.318 for laccase-tetracycline interaction, supporting the ability of the active laccase binding site to interact with these ligands [271].

### 8.2. Ultrasound (Sonolysis) Combined with Enzymatic Degradation for the Degradation of Antibiotics

Sutar and Rathod (2015) worked out the catalytic degradation of Ciprofloxacin (CIP) but using Laccase (Lac, E.C. 1.10.3. 2) in a combined process with sonolysis, achieving 51% degradation under specific conditions (0.02% (w/v) enzyme loading; 60 °C; 75 W input power; 22 kHz frequency; 50% duty cycle and 200 rpm agitation); exceeding the degradation obtained in the conventional process (16%) and reducing the treatment time [272].

Chakma et al. (2020) investigated the links between enzymatic (Horseradish Peroxidase (HRP, EC: 1.11.1.7) coupled with ultrasound processes for Ciprofloxacin (CIP) degradation and found that ultrasound and cavitation improve the degradation efficiency by increasing the interaction between enzyme and organic molecules (kinetic energy). In this sense, Ciprofloxacin (CIP) is enzymatically transformed, so there is no significant improvement in the sono-enzymatic process at which the highest degradation rates at optimum conditions (pH 7.0 and 25 °C) were 68.41%. In contrast, the intermediates, such as acetic acid, ethyl alcohol and other products with -OH groups generated during the sono-enzymatic reaction are less toxic than those formed in the enzymatic process; this means that ultrasound favours mineralization by degrading the intermediates formed during enzymatic treatment [196].

## 9. Conclusions

Antibiotics should be utilised by veterinarians- or physicians-prescription either for metaphylaxis or prophylaxis; however, the indiscriminate use as a growth factor in animals (a prohibited activity in several countries) and by self-medication in humans generates a great effect on the environment and favours the increase and dissemination of antibiotic-resistant microorganisms. As this review shows, the chemical nature of many antibiotics facilitates their excretion through urine or faeces, both in animals and humans. The use of antibiotics is indispensable, despite many international organisations speaking out in favour of their controlled use, many countries are not complying with the rules and continue with inadequate practices, such as the use of antibiotics exclusively for humans in animals, their use as growth factors, biocides in crops, the lack of adequate treatment of wastewaters in pharmaceutical plants, senior residences, farms and hospitals, the use of animal manure as part of biofertilisers and the use of antibiotics as a supplement in animal nutrition, among others.

It is clear that the majority of the antibiotics are recalcitrant and toxic to the environment as they can trigger eutrophication leading to water pollution and nutrient imbalance, and the exposition of microorganisms to the sublethal concentrations of antibiotics also press antibiotic resistance, leading irremediably to the transmission of resistant microorganisms and consequently therapeutic failure in humans and animals.

Many physical and chemical procedures have shown to be effective and improve the management of wastewaters containing antibiotics residues, but the treatment of these wastewaters using enzymes (including laccases) is very promising and appears to be less costly.

## Figures and Tables

**Table 1:** Critical importance and high importance antibiotics, according to WHO.

Structure	Target	Antibiotic	Abbreviations in This Paper	Use	
			CLSI	H	A
Critical Importance Antibiotics					
Aminoglycosides					
Aminoglycosides consist of several cyclitol rings in their structure; usually, three and five sugars are linked by glycosidic bonds. Amino and hydroxyl groups are attached to the rings providing the chemical properties of the compound [23].	They alter cell membrane permeability and also inhibit protein synthesis by binding to the 30s ribosomal subunit [23].	Aminoglycosides + 2 Deoxystreptamine			
		Amikacin	AN	X	
		Paromomycin	PLZ	X	
		Streptomycin	STR	X	X
		Gentamicin	GM	X	X
		Kanamycin	K	X	X
		Netilmicine	NET	X	
		Tobramycin	TM	X	X
		Apramycin	APR		X
		Dihydrostreptomycin	DST		X
		Plazomycin	PLZ	X	
		Neomycin	NEO	X	X
		Isepamycin	ISE	X	
		Arbekacin	ABK	X	
		Fortimicin A	FM-A	X	
		Bekanamycin	AKM	X	
		Dibekacin	DKB	X	
		Ribostamycin	RIB	X	
Ansamycins					
Contain an aliphatic chain connecting the two ends of a naphthoquinone core [24]	Binds to the β subunit of RNA polymerase, inhibiting its activity [24].	Rifampicins			
		Rifaximin	RFP	X	
		Rifampicin	RIF	X	
		Rifapentine	RPT	X	
		Rifabutin	RFB	X	
β-lactams					
The β-lactam ring chemically defines this class of antibiotics. This ring is bound to other radicals. The association of different types of linear chains modifies the properties of the compound and the different groups of β-lactam antibiotics are formed [17,25]. Their structure consists of a five-membered unsaturated ring fused to an β-lactam ring. Carbapenems differ from penicillins by the C2-C3 double bond and the carbon in place of the sulphur at C1. Additionally, carbapenems have a trans-1-hydroxyethyl substituent in place of the acylamino substituent on the β-lactam ring [26].	They act by two mechanisms: inhibition of wall synthesis and induction of bacterial autolysis. Transpeptidase enzymes (E.C. 3.4.16.4) are involved in the last stage of wall synthesis, linking the bonds of the peptidoglycan chains. The β-lactam ring is structurally similar to the region of the pentapeptide to which transpeptidases bind, thus the ring binds to the enzymes inhibiting cell wall formation. In addition, β-lactam activate an endogenous autolysin that degrades the peptidoglycan [17,25].	Carbapenems			
		Doripenem	DOR	X	
		Ertapenem	ETP	X	
		Meropenem	MEM	X	
		Imipenem	IPM	X	
		Panipenem	PAPM	X	
		Faropenem	FRPM	X	
		Biapenem	BPM	X	
The chemical structure of cephalosporins comes from 7-cephalosporanic acid. Cephalosporins structure are a fusion of a two-ring system of -lactam-3-dihydrothiazine, known as 7-aminocephalosporanic acid (7-ACA), and vary in their side-chain substitutions at C3 (R2) and C7 [27].		1, 2, 3,4 and 5 Generation Cephalosporins			
		Cefditoren	CDN	X	
		Cefmenoxime	CMX	X	
		Cephpyrome	CPO	X	
		Ceftriaxone	CRO	X	
		Cefoperazone	CFP		X
		Cefquinome	CEQ		X
		Cefotaxime	CTX	X	
		Ceftazidime	CAZ	X	
		Cefetameta	CAT	X	
		Cefpodoxime	CPD	X	
		Ceftibuten	CBT	X	
		Cefdinir	CDR	X	
		Cefepime	FEP	X	
		Cefixime	CFM	X	
		Ceftaroline fosamil	CPT	X	
		Ceftiofur	CFT		X
		Cefovecin	CFO		X
		Cefaclor	CEC	X	
		Cefadroxil	CFR	X	X
		Cephalexin	CN	X	X
		Cephalonium	CFL		X
		Cephapirin	CAP	X	X
		Cephalotin	CF	X	X
		Cefazolin	CZ	X	X
		Cefoxitin	FOX	X	
		Cefprozil	CPR	X	
		Cefuroxime	CXM	X	X
		Loracarbef	LOR	X	
		Cefotetan	CTT	X	
		Cephradine	BAN	X	
		Tazobactam	TZB	X	
		Cefcapene	CFPM	X	
		Cefodizime	CDZM	X	
		Cefoselis	CFSL	X	
		Cefozopran	ZOP	X	
		Cefsulodin	CFS	X	
		Ceftizoxime	ZOX	X	
		Ceftobiprole	BPR	X	
		Ceftozolane	CTZ	X	
		Latamoxef	LMOX	X	
		Cephacetrile	CEC	X	
		Cephaloridine	CPH	X	
		Cefamandol	CFM	X	
		Cefatrizine	CTZ	X	
		Cefazedone	CFZD	X	
		Cefbuperazone	CFB	X	
		Cefmetazol	CMZ	X	
		Cefminox	CMNX	X	
		Cefonicid	CID	X	
		Ceforanide	CFR	X	
		Cefotiam	CTM	X	
		Cefroxadine	CXD	X	
		Ceftezole	CTZ	X	
		Flomoxef	FMOX	X	
Monobactams have a sulphonic acid group on the nitrogen at the N-l position; the sulphonic acid activates the l3-lactam ring and thus acetylates the transpeptidase enzymes [28].		Penicillin monobactam			
		Aztreonam	ATM	X	
		Carumonam	CAR	X	
The basic structure of penicillin (6-aminopenicillanic acid) consists of a thiazolidine ring, an attached p-lactam ring and a side-chain [17,25].		Antipseudomonal penicillin			
		Carbenicillin	CB	X	
		Ticarcillin	TIC	X	X
		Piperacillin	PIP	X	
		Sulbenicillin	SBPC	X	
		Azlocillin	AZ	X	
		Carindacillin	CIPC	X	
		Mezlocilin	MEZ	X	
		Sultamicillin	SBTPC	X	
		Hetacillin	HET	X	
		Amdinocillin	MEC	X	
		Ampicillin	AM	X	
		Talampicillin	AMX	X	X
		Azidocillin	AZD	X	
		Bacampicillin	B	X	
		Epicillin	EP	X	
		Temocillin	TEM	X	
		Methampicillin	MTP	X	
		Pivampicillin	PMPC	XX	
		Amoxicillin (Clavulanic acid)	AMC	X	X
Glycopeptides and Lipoglycopeptides					
Consist of a central heptapeptide core to which amino acid residues and sugars are attached [29].	In Gram-positive bacteria, Glycopeptides and lipoglycopeptides bind to the D-alanyl-D-alanine terminus at the carboxy-terminal end of bacterial wall precursor peptides, thus blocking peptidoglycan synthesis [24,29].	Telavancine	TLV	X	
		Teicoplanin	TEC	X	
		Vancomycin	VA	X	
		Avoparcin	AV	X	
		Dalbavancine	DAL	X	
		Oritavancine	ORI	X	
		Ramoplanin	RAM	X	
Glycyliclinas					
Structurally similar to the tetracyclines, it has a central structure of four carbocyclic rings, with a t-butylglycylamide substitution at position 9 of the minocycline that confers a broad spectrum of activity [24].	Inhibits protein synthesis by reversibly binding to the 30S subunit of the bacterial ribosome, blocking the entry of the aminoacyl tRNA into the A site of the ribosome, thus preventing amino acid incorporation and subsequent elongation of peptide chains [24].	Tigecycline	TGC	X	
Lipopeptides					
A cyclic molecule with 13 amino acids, ten of which are part of the cyclic structure, and the remaining three make up a side chain with an N-decanoyl residue [24].	They insert into the membrane bilayer causing its depolarisation, with a strong loss of potassium ion leading to cell death; a side-chain bearing an N-decanoyl residue [24].	Daptomycin	DAP	X	
		Colistin	CL	X	
		Polymyxin B	PB	X	X
Macrolides					
Are a lactonic ring with 14 to 16 carbons, bound to an aminated sugar [24].	Binds to sequences of the 23S rRNA domain V, which is part of the 50S subunit, preventing elongation of the peptide chain by blocking the polypeptide exit tunnel and thus dissociating the peptidyl-RNA complex from the ribosome [24].	Azithromycin	AZM	X	X
		Gamithromycin	GM	X	X
		Josamycin	JM	X	X
		Tulathromycin	TUL		X
		Tylvalosin	TVN		X
		Tylosin	TLY		X
		Tilmicosin	TMS		X
		Midecamycin	MDM	X	
		Dirithromycin	DTM	X	
		Rokitamycin	RKM	X	
		Roxithromycin	RXT	X	
		Clarithromycin	CLR	X	
		Spiramycin	SP		X
		Fidaxomicin	FDX	X	
		Erythromycin	E	X	
		Telithromycin	TEL	X	X
		Fluoroerythromycin		X	
		Kitasamycin	KIA	X	
		Oleandomycin	OL	X	
		Tildispyrosine	TD	X	
		Troleandomycin	TAO	X	
		Quinupristin-dalfopristin	SYN	X	
		Pristinamycin	PT	X	
		Virginiamycin	VM		X
		Solithromycin	SOL	X	
		Cethromycin	CET	X	
Oxazolidinones					
Oxazolidinone consists of a ring with three carbon atoms, an oxygen atom at position one and a nitrogen atom at position three [30].	The antibiotic binds to the 50S subunit, affecting protein synthesis, and also inhibits the initiation complex by binding to the 70S subunit. The D-ring of the tedizolid contributes to the presence of additional hydrogen bonds that provide further interactions between the tedizolid and the bacterial ribosome; therefore, the drug is more potent [30].	Radezolid	RAD	X	
		Linezolid	LZD	X	
		Cadazolid	CDZ	X	
		Tedizolid	TZD	X	
Phosphomycins					
It is cis-1,2-epoxypropylphosphonic acid (-), a simple, water-soluble molecule with only three carbon atoms and no nitrogen. The carbon atom is bonded to the phosphorus atom without an intermediate oxygen bridge. The antimicrobial activity is due to the epoxy bond [24].	Fosfomycin enters the membrane via two permeases (E.C. 3.1.3.9); the inducible D-glucose-6-phosphate transport system and the L-α-glycerophosphate system. The antibiotic competes with the substrate of the enzyme UDP-N-acetylglucosamine-3-O-enolpyruvyltransferase (MurA) (E.C. 2.5.1.7), an enzyme that catalyses the first stage of peptidoglycan heteropolymer biosynthesis [24].	Fosfomycin	FOS	X	X
Quinolones and Fluoroquinolones					
They interfere with DNA synthesis inducing cell death and penetrating the wall through porins to interact with two enzymes, the DNA gyrase (E.C. 5.6.2.2) and topoisomerase IV (E.C. 5.99.1.2), both responsible for DNA supercoiling [24].	Quinolones and fluoroquinolones interfere with DNA synthesis, inducing cell death. They penetrate the wall through porins and interact with two enzymes; DNA gyrase (E.C. 5.6.2.2) and topoisomerase IV, (E.C. 5.99.1.2) responsible for DNA supercoiling. [24].	Pefloxacin	PEF	X	
		Besifloxacin	BES	X	
		Delafloxacin	DLX	X	
		Gemifloxacin	GEM	X	
		Nadifloxacin		X	
		Nalidixic acid	NAL	X	
		Ozenoxacin	OZN	X	
		Oxolinic acid	OA	X	
		Enrofloxacin	ENR		X
		Difloxacin	DIF		X
		Pradofloxacin	PARA		X
		Moxifloxacin	MXF	X	X
		Levofloxacin	LVX	X	X
		Ibafloxacin			X
		Flumequine	FLU		X
		Marbofloxacin	MAR		X
		Orbifloxacin	OBFX		X
		Rufloxacin	RFX	X	X
		Ciprofloxacin	CIP	X	X
		Norfloxacin	NX	X	
		Gatifloxacin	GAT	X	
		Ofloxacin	OFL	X	
		Lomefloxacin	LOM	X	
		Enoxacin	GRN	X	
		Grepafloxacin	GRX	X	
		Pazufloxacin	PZFX	X	
		Pipemidic acid	HPPA	X	
		Prulifloxacin	PUFX	X	
		Rosoxacin	RSX	X	
		Sitafloxacin	STFX	X	
		Sparfloxacin	SPX	X	
		Termafloxacin	TEM		
Phenicols					
Phenicols feature a p-nitrophenyl group and an N-dichloroacetyl substituent attached to a three-carbon chain [24].	Prevents peptide bond formation by binding to the L16 protein located in the 50S subunit of the ribosome, which mediates tRNA binding to peptidyl transferase (E.C. 2.3.2.12) [24].	Chloramphenicol	CHL	X	X
		Thiamphenicol		X	X
		Florfenicol	FLO		X
Lincosamides					
Lincomycin consists of an amino acid attached to an amino sugar; clindamycin differs structurally due to the substitution of a chlorine atom by a hydroxyl group and the inversion of carbon [24].	It binds to sequences of rRNA 23S domain V, which is part of the 50S subunit of the ribosome, preventing the elongation of the peptide chain by blocking the polypeptide exit tunnel, and thus the peptidyl-RNA complex dissociates from the ribosome [24].	Clindamycin	CM	X	X
		Lincomycin	LIN	X	X
		Pirlimycin	PIR		X
Pseudomonic Acids					
It consists of a 9-hydroxy-nonanoic acid chain, some spatial similarity in structure to the amino acid isoleucine [31].	DNA gyrase and topoisomerase IV inhibitor [32]. Binds to the enzyme isoleucyl-tRNA (E.C. 6.1.1.5), preventing the incorporation of isoleucine into proteins [31].	Mupirocin	MUP	X	
Rhinophenazines					
It contains a phenazine core with an alkyl imino (R-imino) group at position two and phenyl substituents at positions 3 and 10 of the phenazine core. The alkyl imino group is essential for antimicrobial activity [33].	Its target is the bacterial respiratory chain and ion transporters. Intracellular redox cycling, involving oxidation of reduced clofazimine, generates antimicrobial reactive oxygen species (ROS) and hydrogen peroxide (H_2_O_2_). Additionally, clofazimine interaction with membrane phospholipids promotes membrane dysfunction and interferes with K^+^ uptake. Both mechanisms result in interference with cellular energy metabolism by disrupting ATP production [33].	Clofazimine	CFZ	X	
Steroidals					
They have a steroid structure (cyclopentanoperhydrophenanthrene ring), and are a tetracyclic triterpenoid [34].	Inhibits protein synthesis by preventing translocation of elongation factor (EF-G) during protein synthesis [34].	Fusidic acid	FUS	X	
Sulphonamides					
They have a similar structure to para-aminobenzoic acid. Contain an aromatic NH_2_ substituent at the para position of the benzene ring. Contain a substituent at the ortho- and meta-position of the benzene ring. Additionally, it contains a double substituent at position N1 and a sulphonamide group on the benzene ring [24].	They are competitive p-aminobenzoic acid (PABA) antagonists, binding to the enzyme tetrahydropteroic acid synthetase (E.C 6.3.2.17) inhibiting folic acid synthesis [24].	Sulfamethoxazole	SMX	X	X
		Sulphadimethoxine	SDM		X
		Sulphadiazine	SDZ	X	X
		Sulfamerazine	SMZ		X
		Sulphanilamide	SA		X
		Sulfathiazole	STZ	X	X
		Sulfadimidine = Sulfamethazine	SM2		X
		Sulfamethizole	SMZ	X	X
		Sulfisoxazole	FIS		X
		Trimethoprim	SXT	X	X
		Brodimoprim	BDM	X	
		Formosulfathiazole		X	
		Iclaprim	ICL	X	
		Phthalylsulfathiazole	PA	X	
		Sulphaisodimidine	SU	X	
		Sulphalene		X	
		Sulfamazone	SZO	X	
		Sulfamethoxypyridazine	SP	X	
		Sulphamethomidine	SM	X	
		Sulfamethoxydiazine	SMD	X	
		Sulphametrol	SMT	X	
		Sulfamoxol	SMO	X	
		Sulfaperin	SFL	X	
		Sulfafenazol	SPZ	X	
		Sulphapyridine	SP	X	
		Sulfatiourea	SFTu	X	
		Tetroxoprim	TXP	X	
Tetracyclines					
Composed of a linear fused tetracyclic fused core to which several functional groups are attached (Chlorine (Cl), Hydrogen (H) dimethylamine (N(CH_3_)_2_, methyl (CH_2_), methylenes (CH_3_) hydroxyl (OH) [35].	It binds to the 30S subunit of the bacterial ribosome and prevents the binding of the aminoacyl tRNA, disrupting the incorporation of amino acids into the growing peptide [35].	Chlortetracycline	CTC	X	X
		Demeclocycline	DMC		X
		Omadacycline	OMC		X
		Lymecycline	LYME	X	
		Eravacycline	ERV		X
		Doxycycline	DO	X	X
		Oxytetracycline	OXT	X	X
		Rolitetracycline	RTC		X
		Tetracycline	TE	X	X
		Minocycline	MI	X	
		Clomocycline	CLM	X	
		Penimepicycline	PNM	X	
		Methacycline	ME	X	

H: Humans. A: Animals.

**Table 2:** Types of treatments and techniques for antibiotics degradation, summary of degradation or removal percent.

Types of Antibiotic Degradation	Techniques	Process or Materials	Antibiotics Reported	Degradation/Removal	References
Biotic	Hydrolysis	Lake sediment.	Cephradine (BAN), Cefuroxime (CXM), Ceftriaxone (CRO), Cefepime (FEP).	Reporting removal of Ceftriaxone disodium (CRO) of 3% and Cefotiam dihydrochloride (CTM) of 7% in 28 d.	[155]
	Microbial degradation	Bacterial suspensions of poultry manure and soil that produce humic acids.	Tetracycline (TE), Oxytetracycline (OXT), Chlortetracycline (CTC).	Removal between 88 and 75% in 15 min.	[218]
	Degradation attributed to a co-metabolic process	The strain was isolated from a reactor used for the treatment of aquaculture effluent.	Oxytetracycline (OXT), Ciprofloxacin (CIP).	Oxytetracycline (OXT) between 90.3 and 97.4 and Ciprofloxacin (CIP) were unable to degrade.	[159]
Chemical	Heat activated of Persulfate	Aqueous solution at different pH.	Penicillin G (PG).	At pH 5 was removal 82.6 and at higher pH, the removal decreased.	[219]
	Electrochemical oxidation	EC reactor with a built-in platinum counter electrode and Roxy potentiostat.	Ciprofloxacin (CIP), Norfloxacin (NX), Ofloxacin (OFL).	Reporting removal of Ciprofloxacin (CIP) of 90%, Norfloxacin (NX) of 62% and Ofloxacin 97.3%.	[220]
	Fenton process	The reaction was improving with H_2_O_2_, promoting catalytic production.	Thiazole sulphate, Tylosin (TLY), Ciprofloxacin (CIP), Amoxicillin (AMC) Cloxacillin (CLO), Tetracycline (TE).	Reporting removal between 77 and 97.1%.	[221]
Physical	Adsorption	Biochar of waste sludge.	Tetracycline (TE), Sulfamethazine (SM2)	Weak adsorption using biochar could be overcome with the use of peroxymonosulfate.	[222]
		ZSM-5 zeolite and zeolites nanocrystals.	Ciprofloxacin (CIP).	Removal between 54 and 90% according to material, time and antibiotic concentration.	[223]
	Temperature	Increased temperature with incubation	Sulphonamide (SUL).	Minimum temperature of 60% removing between 78.1 and 98.3 on different sulphonamide antibiotics.	[195]
	Photodegradation	It was evaluated under a 125 W UV A-B-C (200–600 nm) irradiation.	Ciprofloxacin (CIP), Ofloxacin (OFL).	There is no high evidence of degradation of antibiotics by exposure to UV light.	[224]
Physico-chemical	Plasma treatment	Plasma reactor in coaxial configuration operated in pulsed mode.	Oxacillin (OX), Amoxicillin (AMC), Ampicillin (AM).	Oxacillin and Amoxicillin (AMC) had >90% conversion and Ampicillin (AM) was 29%.	[191]
	Photocatalysis	Solarbox 1500 photoreactor produced by an air-cooled xenon lamp.	Ampicillin (AM), Enrofloxacin (ENR), Tylosin (TLY), Vancomycin (VA), Clindamycin (CM), Trimethoprim (SXT), Zetronidazole, Sulfadiazine (SD), Doxycycline (DO), Oxytetracycline (OXT).	The removal of antibiotics by photocatalytic oxidation was between 40 and 100% with the exception of Clindamycin (CM) which was not removed.	[225]
		Quartz reactor with a 20 W lamp and irradiation of 2300 μW/cm^2^.	Ciprofloxacin (CIP), Ofloxacin (OFL).	Removal of ~70%.	[226]
	Advanced oxidation processes (AOP)	Oxidation with agents such as hydrogen peroxide, ozone, titanium dioxide and UV light using semiconducting materials, such as TiO_2_, SnO_2_, CeO_2_, ZnO as catalysts.	Penicillins, Sulphonamides, Phenicols, β-lactams, Tetracyclines, Fluoroquinolones	Reporting removal with different efficiency ranges between 78 and 100%.	[227]

## Data Availability

Data are contained within the article.
